# Boosting the Power of Rare Variant Association Studies by Imputation Using Large-scale Sequencing Population

**DOI:** 10.1093/gpbjnl/qzaf084

**Published:** 2025-09-17

**Authors:** Jinglan Dai, Yixin Zhang, Yuan Gao, Hongru Li, Sha Du, Hao Hong, Dongfang You, Zaiming Li, Ruyang Zhang, Yang Zhao, Zhonghua Liu, David C Christiani, Feng Chen, Sipeng Shen

**Affiliations:** Department of Biostatistics, Center for Global Health, School of Public Health, Nanjing Medical University, Nanjing 211166, China; Department of Biostatistics, Center for Global Health, School of Public Health, Nanjing Medical University, Nanjing 211166, China; Department of Biostatistics, Center for Global Health, School of Public Health, Nanjing Medical University, Nanjing 211166, China; Department of Biostatistics, Center for Global Health, School of Public Health, Nanjing Medical University, Nanjing 211166, China; Department of Biostatistics, Center for Global Health, School of Public Health, Nanjing Medical University, Nanjing 211166, China; Jiangsu Key Lab of Cancer Biomarkers, Prevention and Treatment, Jiangsu Collaborative Innovation Center for Cancer Personalized Medicine, Nanjing Medical University, Nanjing 211166, China; Department of Biostatistics, Center for Global Health, School of Public Health, Nanjing Medical University, Nanjing 211166, China; Department of Biostatistics, Center for Global Health, School of Public Health, Nanjing Medical University, Nanjing 211166, China; China International Cooperation Center of Environment and Human Health, Nanjing Medical University, Nanjing 211166, China; Department of Biostatistics, Center for Global Health, School of Public Health, Nanjing Medical University, Nanjing 211166, China; Department of Biostatistics, Center for Global Health, School of Public Health, Nanjing Medical University, Nanjing 211166, China; Key Laboratory of Biomedical Big Data of Nanjing Medical University, Nanjing 211166, China; Department of Biostatistics, Center for Global Health, School of Public Health, Nanjing Medical University, Nanjing 211166, China; Key Laboratory of Biomedical Big Data of Nanjing Medical University, Nanjing 211166, China; Department of Biostatistics, Columbia University, New York, NY 10027, USA; Department of Environmental Health, Harvard T.H. Chan School of Public Health, Harvard University, Boston, MA 02115, USA; Pulmonary and Critical Care Division, Massachusetts General Hospital, Department of Medicine, Harvard Medical School, Boston, MA 02114, USA; Department of Biostatistics, Center for Global Health, School of Public Health, Nanjing Medical University, Nanjing 211166, China; Jiangsu Key Lab of Cancer Biomarkers, Prevention and Treatment, Jiangsu Collaborative Innovation Center for Cancer Personalized Medicine, Nanjing Medical University, Nanjing 211166, China; China International Cooperation Center of Environment and Human Health, Nanjing Medical University, Nanjing 211166, China; Department of Biostatistics, Center for Global Health, School of Public Health, Nanjing Medical University, Nanjing 211166, China; Jiangsu Key Lab of Cancer Biomarkers, Prevention and Treatment, Jiangsu Collaborative Innovation Center for Cancer Personalized Medicine, Nanjing Medical University, Nanjing 211166, China; Key Laboratory of Biomedical Big Data of Nanjing Medical University, Nanjing 211166, China

**Keywords:** Rare variant, Whole-genome sequencing, Genome-wide association study, Genotype imputation

## Abstract

With the emergence of population-scale whole-genome sequencing (WGS), rare variants can be captured precisely. Studying rare variants explains part of the heritability of complex traits that is overlooked by conventional genome-wide association studies (GWASs). However, the extent to which imputed data can approximate or improve upon the power of WGS data in rare variant association studies remains unclear. Using the UK Biobank WGS data (*n* = 150,119) as the ground truth, we first evaluated the consistency of rare variants in the single-nucleotide polymorphism (SNP) array data imputed using TOPMed or HRC+UK10K reference panel. Imputation quality (average *R*^2^) of the TOPMed-imputed data reached 0.6 even for extremely rare variants with minor allele count ≤ 5. TOPMed-imputed data were closer to WGS data across three ethnic groups, with average Cramer’s V > 0.75. Furthermore, association tests were performed on 45 traits. At the same sample size (*n* = 150,119), neither imputed dataset outperformed WGS data, but the results of the TOPMed-imputed data were more consistent with those of WGS data. When the sample size was increased to 488,377, the number of significant rare variants identified from the TOPMed-imputed data increased by 27.71% for quantitative traits and by approximately 10-fold for binary traits. Finally, we meta-analyzed the association results of SNP array and WGS for lung cancer and epithelial ovarian cancer, respectively. Compared to WGS-based results, more significant variants and genes were identified. Our findings highlight that incorporating rare variants imputed using large-scale sequencing populations can boost the power of rare variant association studies when WGS has limited sample sizes.

## Graphical abstract

**Figure:**
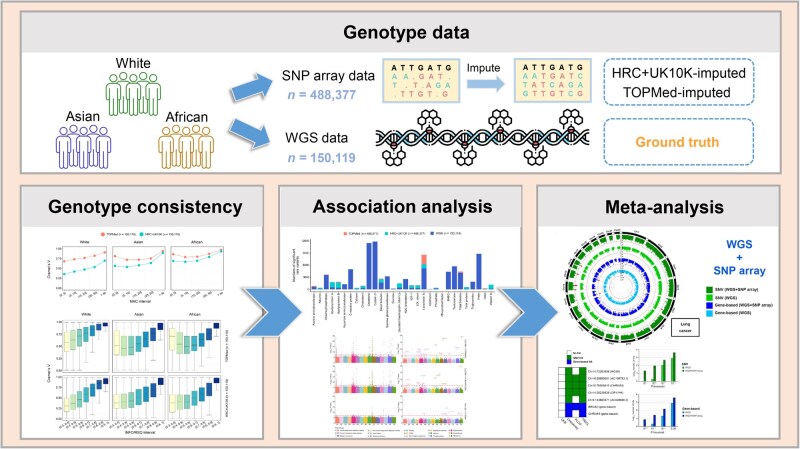

## Introduction

Conventional genome-wide association studies (GWASs) are well-powered to detect thousands of common variants associated with human traits and diseases [1–3]. However, these GWASs underrepresent rare variants due to the limitations of single-nucleotide polymorphism (SNP) arrays [4]. Rare variants tend to have larger effects and behave differently from common variants to explain a fraction of human traits or disease heritability [5,6]. In particular, rare coding germline variants, such as loss-of-function (LoF) and missense variants, exhibit strong functional evidence for deleteriousness [7,8]. With the emergence of next-generation sequencing (NGS) [*e.g.*, whole-genome sequencing (WGS)] based on biobank-level populations, rare variants [minor allele frequency (MAF) < 0.01] in the genome can be accurately captured, rather than being limited to identifying the prespecified few hundred thousand common variants (MAF ≥ 0.01) on a genotyping array [9]. NGS thus provides a valuable opportunity to explore associations between rare variants and complex human traits. Several sequencing-based GWASs aimed at identifying novel variants in cancer and large-scale WGS studies in human traits have benefited from NGS [10–13].

A recent study has shown that population-specific reference genomes can enhance variant calling accuracy in target populations [14]. However, the high cost of deep WGS makes it difficult to obtain the original data of the target population in some large sample studies [15]. Even when high-quality population-based sequencing data are available, the number of complex disease cases is often small, limiting the power to identify rare variants compared to SNP array-designed case-control studies. For example, the UK Biobank (UKB) has WGS data from approximately half a million individuals (*n* ≈ 490,000) [16–18], but the number of incident cases for most cancers is less than 5000. SNP array is still a cost-effective and major approach for human genomics exploration thus far, such as the UCLA ATLAS Community Health Initiative [19], FinnGen [17], and various case-control GWAS consortia [20]. Nevertheless, unlike sequencing data, SNP array-based genotyping captures only a small fraction of genetic variation across the genome. Thus, a more effective way to power association analysis is to capture undetected rare variants by imputation using the external, high-quality, large-scale sequencing reference panels [15,21], such as the UK10K [22], the Haplotype Reference Consortium (HRC) [23], and the Trans-Omics for Precision Medicine (TOPMed) [24]. For example, 290 million (∼  97%) variants had MAF < 0.01 in TOPMed, which might be strong candidates for association analyses [24]. Leveraging such large-scale reference panels in genotype imputation to infer untyped variants from sequencing data can capture a broader spectrum of rare variants, enhance genomic coverage, and improve the statistical power of GWAS [25]. However, when extending the imputation approach to imputed ultra-rare variants [*e.g.*, minor allele count (MAC) ≥ 3] in GWASs, the accuracy and the impact of different reference panels remain uncertain.

Most prior studies have focused on either the superiority of using rare variant imputation for analyses or the accuracy and efficiency of the imputation algorithms themselves. Some studies have shown that incorporating genotype imputation (especially for rare variants) into GWAS can provide more information about previously unreported rare variants and facilitate research on specific diseases or traits [26–28]. Pistis et al. investigated multiple factors that may affect the performance of rare variant genotype imputation by using different reference panels [29]. Recently, several studies focused on the imputation accuracy and computational efficiency of various phasing and imputation tools (*e.g.*, SHAPEIT5, IMPUTE, and Minimac4) [30–32].

Although genotype imputation has been the workhorse of GWAS over the past decades [20,33], rare variants with MAF < 0.01 are generally filtered out due to the limited size of earlier reference panels (*e.g.*, 1000 Genomes Project [34] and the International HapMap Project [35]). Consequently, prior studies evaluating rare variants from SNP array data without imputation were restricted to small-scale associations for specific diseases [36]. With the emergence of large-scale sequencing panels, only a limited number of studies have systematically evaluated how closely rare variants in SNP array data imputed using these panels approximate real WGS data and have assessed their performance in rare variant association analyses. To date, limited comprehensive large-scale studies have evaluated the imputation quality of rare variants and their correlation with WGS. Furthermore, few studies have systematically compared rare variant association results across quantitative and binary traits between imputed and WGS data. Therefore, it remains unclear whether the power achievable with imputed data can approach or exceed that of WGS data in rare variant association studies under certain conditions (such as varying sample sizes or trait types).

Here, we leveraged the WGS data of 150,119 individuals from UKB as the ground truth to evaluate the coverage and accuracy of rare variant imputation using HRC+UK10K and TOPMed reference panels. We then performed association tests for both WGS data and imputed variants with 30 biochemical markers and 15 complex diseases in two sample size scales (*n* = 150,119 and *n* = 488,377). Finally, we performed meta-analyses on lung cancer (LC) and epithelial ovarian cancer (EOC). By combining association results from the WGS data and diverse SNP array datasets across different cohorts, we identified additional rare variants and genes implicated in these two cancers.

## Results

### Landscape of rare variant imputation

**Figure 1:**
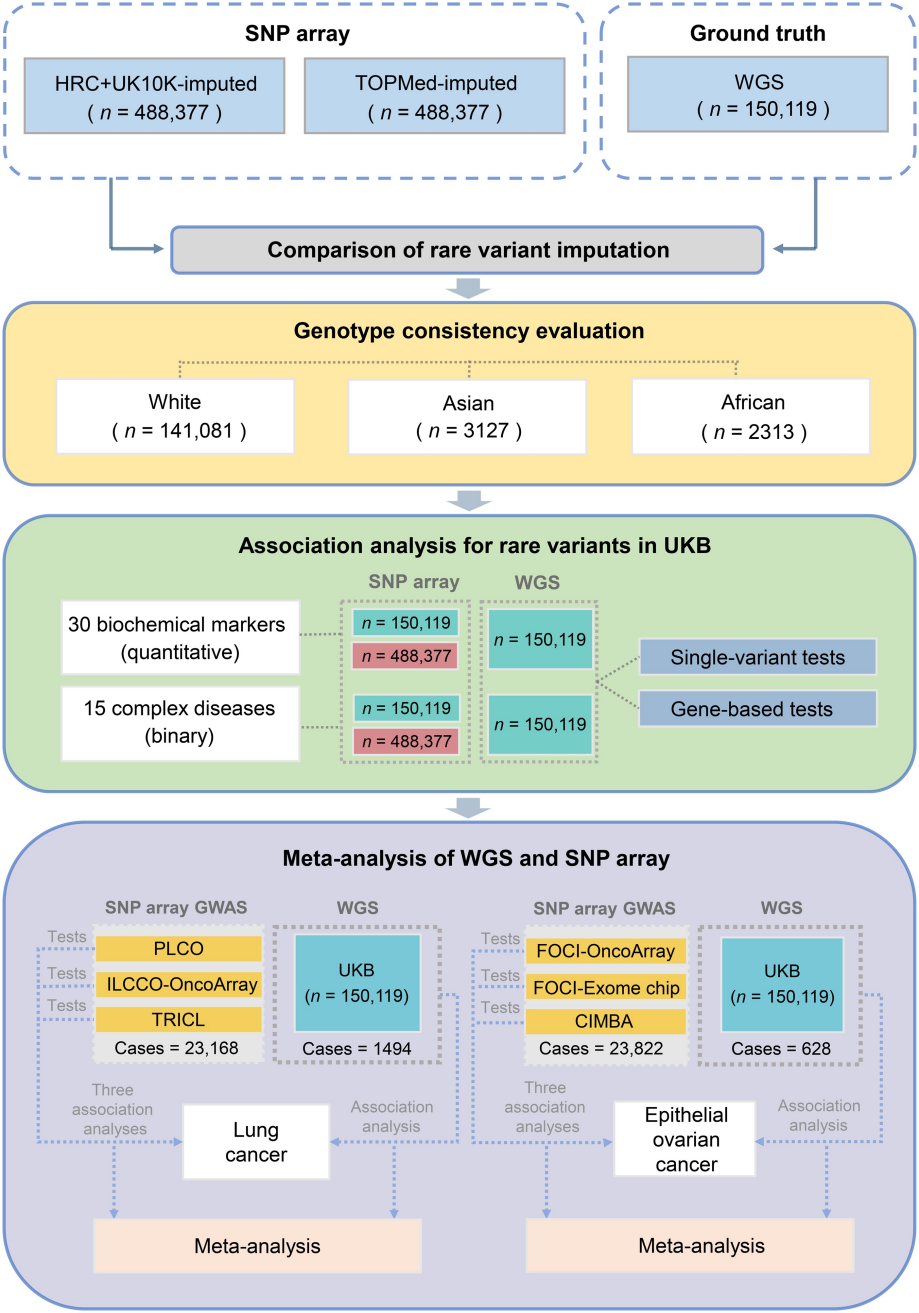
Study workflow The schematic of the analytical pipeline summarizes the main steps for conducting a comparative study using WGS as the ground truth. Step 1: rare variant comparison for the two imputed datasets (TOPMed-imputed and HRC+UK10K-imputed) and the WGS data. Step 2 (yellow block): genotype consistency evaluation across three ethnic groups. Step 3 (green block): single-variant association tests and gene-based association tests on 45 traits in each dataset. Step 4 (purple block): association analyses on three SNP array-imputed datasets and WGS data of lung cancer and epithelial ovarian cancer, respectively, followed by cancer-specific meta-analysis to integrate the association analysis results of their respective cohorts with the association analysis results of the WGS data. SNP, single-nucleotide polymorphism; WGS, genome-wide sequencing; UKB, UK Biobank; PLCO, Prostate, Lung, Colorectal, and Ovarian cancer screening trial; ILCCO-OncoArray, International Lung Cancer OncoArray Consortium; TRICL, Transdisciplinary Research in Cancer of the Lung; FOCI, Follow-up of Ovarian Cancer Genetic Association and Interaction Studies; FOCI-OncoArray, Follow-up of Ovarian Cancer Genetic Association and Interaction Studies OncoArray Consortium; FOCI-Exom chip, Follow-up of Ovarian Cancer Genetic Association and Interaction Studies Exome genotyping array components; CIMBA, Consortium of Investigators of Modifiers of BRCA1/2.

**Figure 2:**
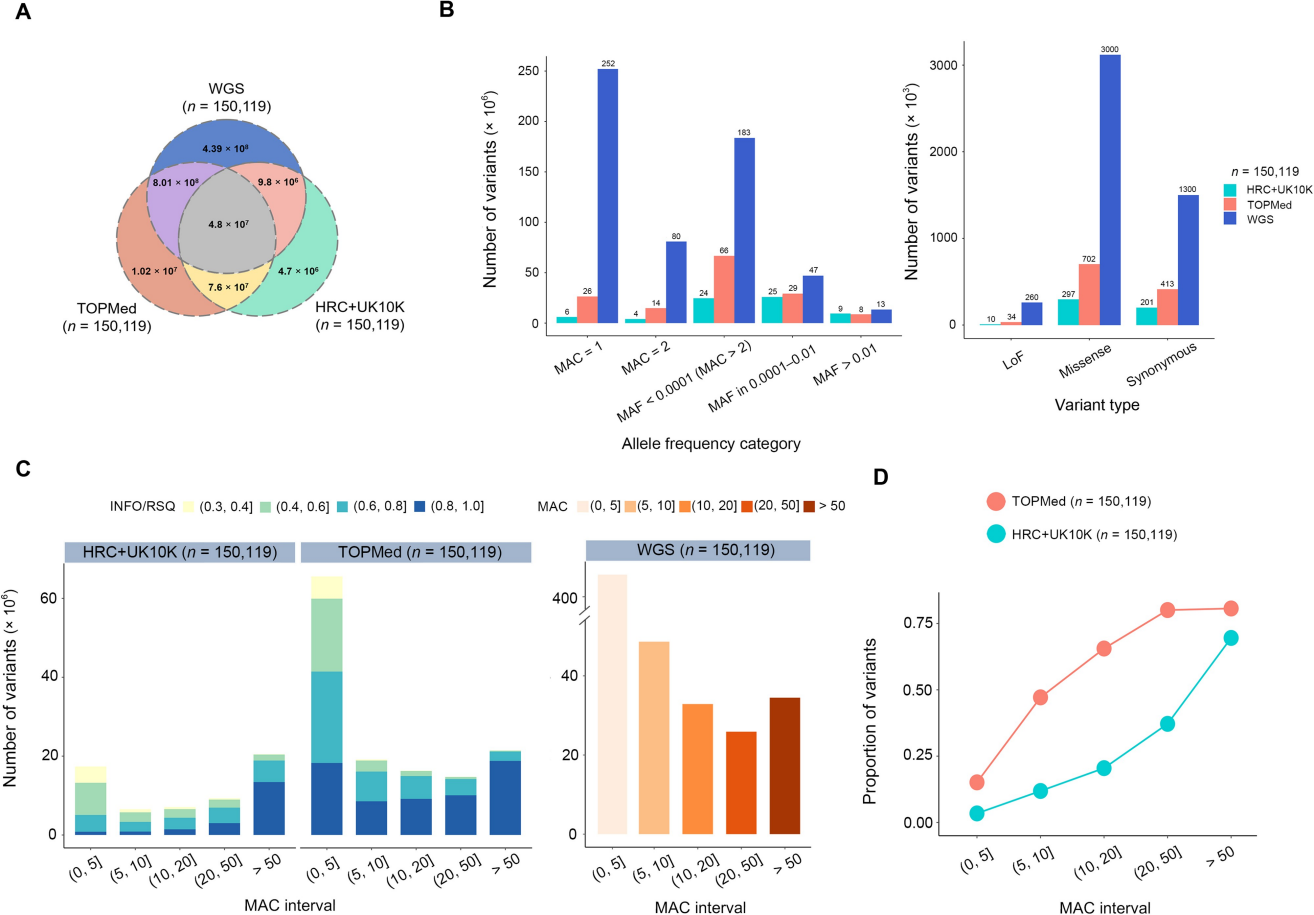
Landscape of rare variant imputation Only variants with INFO/RSQ > 0.3 were included in the statistics. **A**. Venn diagram showing the overlap of variants among TOPMed-imputed, HRC+UK10K-imputed, and WGS data (each *n* = 150,119). **B**. Bar charts illustrating the number of variants classified by allele frequency category (left) and variant type (right) across datasets. **C**. Left: distribution of variants in different INFO/RSQ bins across five MAC intervals for the two imputed datasets. Right: distribution of variants across five MAC intervals for the WGS dataset. **D**. Line chart showing the recovery proportion of WGS variants in different imputed datasets across five MAC intervals. INFO, information measure; RSQ, MACH *R*^2^; MAC, minor allele count; MAF, minor allele frequency; LoF, loss-of-function.

We first analyzed the genetic variants in the WGS data of 150,119 UKB individuals and in the matched genotype data imputed using the HRC+UK10K and TOPMed reference panels (**Figure 1**, Figure S1). The overlap of variants between the WGS and imputed data is shown in **Figure 2**A. TOPMed-based imputation captured a substantially larger number of genetic variants, including 22.2% of the single-nucleotide variants (SNVs) in the WGS data, which was higher than HRC+UK10K (10.0%). The TOPMed-imputed data contained 40 million singleton (MAC = 1) and doubleton (MAC = 2) variants, which were 4-fold of the HRC+UK10K-imputed data but fewer than the 332 million such variants in the WGS data. Additionally, the TOPMed-imputed data contained 66 million ultra-rare variants (MAF < 0.0001 and MAC > 2), a count equivalent to 36.3% of the ultra-rare variants identified in the WGS data. In contrast, the HRC+UK10K-imputed data only contained 24 million ultra-rare variants, far fewer than those in the TOPMed-imputed data. For variants with 0.0001 ≤ MAF ≤ 0.01 and common variants (MAF > 0.01), the difference in variant count between the two imputed datasets was significantly reduced; however, both contained fewer such variants than the WGS data (Figure 2B; Table S1).

In addition, we collected the annotations of protein-coding variants from gnomAD and described the variant landscapes of the two matched imputed datasets based on variant type classification. Specifically, the TOPMed-imputed data contained 34,661 LoF variants, 413,571 synonymous variants, and 702,141 missense variants, with the total number of protein-coding variants approximately 2.2-fold greater than that from the HRC+UK10K-imputed data (Figure 2B).

For imputed data, post-imputation quality control is an important step based on information measure/MACH *R*^2^ (INFO/RSQ) filtering. Figure 2C shows the variant counts in different INFO/RSQ bins across five MAC intervals for the two matched imputed datasets, as well as the total variant counts within the corresponding MAC intervals for the WGS data. In the smallest MAC interval (MAC ∈(0, 5]), the number of variants with INFO/RSQ of 0.8–1.0 in the TOPMed-imputed data ($n_{\mathrm{snv}}\;$= 8,537,429) was approximately 9-fold more than that in the HRC+UK10K-imputed data ($n_{\mathrm{snv}}$ = 819,134), but only about 20% of the variant count in the WGS data ($n_{\mathrm{snv}}$ = 421,848,246). In terms of overall rare variant imputation, the average INFO/RSQ of the TOPMed-imputed data was 0.79, while that of the HRC+UK10K-imputed data was only 0.66 (Table S2).

To assess how closely the imputed data approximate the WGS data, we calculated the proportion of WGS variants recovered by imputation in each MAC interval (Figure 2D). As expected, this recovery proportion increased with MAC. When MAC ∈(10, 20], the TOPMed-imputed data captured approximately 65% of the WGS variants, whereas the HRC+UK10K-imputed data captured only approximately 20% (Figure 2D; Table S3).

### Evaluation of genotype consistency between WGS and imputed data

**Figure 3:**
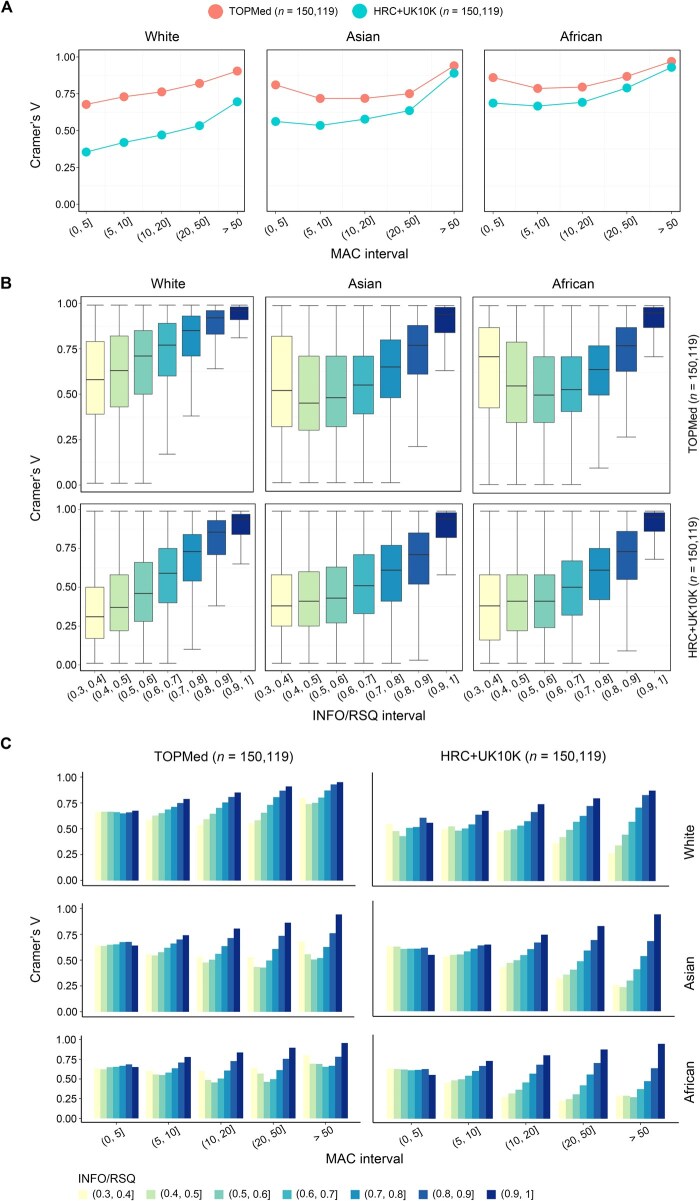
Consistency evaluation of the imputed datasets across ethnic groups **A**. Cramer’s V between imputed data and WGS data in different MAC intervals across three ethnic groups. **B**. Box plots showing the relationships between the INFO/RSQ of imputed data and Cramer’s V. **C**. Bar plots showing the relationships between Cramer’s V and different combinations of MAC and INFO/RSQ across three ethnic groups. The *x*-axis represents different MAC intervals, and the *y*-axis represents Cramer’s V. Each bar corresponds to an INFO/RSQ interval.

We used WGS data as the ground truth to conduct correlation analyses on the two imputed datasets (*n* = 150,119, matched to WGS data) to evaluate the consistency of rare variants across three ethnic groups (White, Asian, and African) (Table S4). TOPMed-imputed data showed better imputation performance, more closely approximating WGS data than HRC+UK10K-imputed data in all MAC intervals. This was reflected by the average Cramer’s V for TOPMed-imputed data, which exceeded 0.75 in all three ethnic groups. Even for extremely rare variants with MAC ∈(0, 5], the Cramer’s V for TOPMed-imputed data exceeded 0.60 in all ethnic groups (**Figure 3**A; Table S5). In addition, we used Pearson’s squared correlation (Pearson’s *r*^2^) as an additional metric for correlation analyses. The trend of Pearson’s *r*^2^ increasing with MAC in different ethnic groups was consistent with that observed for Cramer’s V. Under this metric, the imputation performance of TOPMed-imputed data remained closer to WGS, with the average Pearson’s *r*^2^ of all MAC intervals reaching at least 0.70 in each ethnic group (Figure S2; Table S5).

We then investigated the relationship between imputation quality (INFO/RSQ) and Cramer’s V, by calculating Cramer’s V for variants with INFO/RSQ in each given interval (Figure 3B). As expected, Cramer’s V increased with INFO/RSQ in all three ethnic groups, indicating that variants with higher imputation quality were closer to WGS data. Surprisingly, even within the same INFO/RSQ bin, the consistency of Cramer’s V in TOPMed-imputed data was higher than that in HRC+UK10K-imputed data. Moreover, the TOPMed-imputed data showed a stable consistency (Cramer’s V > 0.50 across all INFO/RSQ bins) even when the imputation quality was relatively low (INFO/RSQ ∈(0.3, 0.4]) (Table S6).

In addition, we observed changes in Cramer’s V when combining different MAC intervals with INFO/RSQ bins (Figure 3C). For the two imputed datasets in each ethnic group, it could be seen from the combination of MAC ∈(10, 20] and INFO/RSQ ∈(0.5, 0.6] that Cramer’s V was proportional to MAC and INFO/RSQ. Furthermore, similar to the results when MAC and INFO/RSQ were considered separately, the performance of TOPMed-imputed data was better than that of HRC+UK10K-imputed data across all combinations of MAC and INFO/RSQ. Therefore, rare variants imputed from TOPMed exhibited promise for subsequent association analyses.

### Rare variant association analysis in 150,119 UKB participants

To explore the performance of different imputed data in rare variant (MAF < 0.01) association analyses and to compare them with WGS data, we first carried out association analyses for 45 traits (30 biochemical markers and 15 complex diseases) on the White population (matched to 150,119 participants with WGS data) using the two imputed datasets, respectively, and conducted identical analyses on the WGS data for apples-to-apples comparisons.

**Figure 4:**
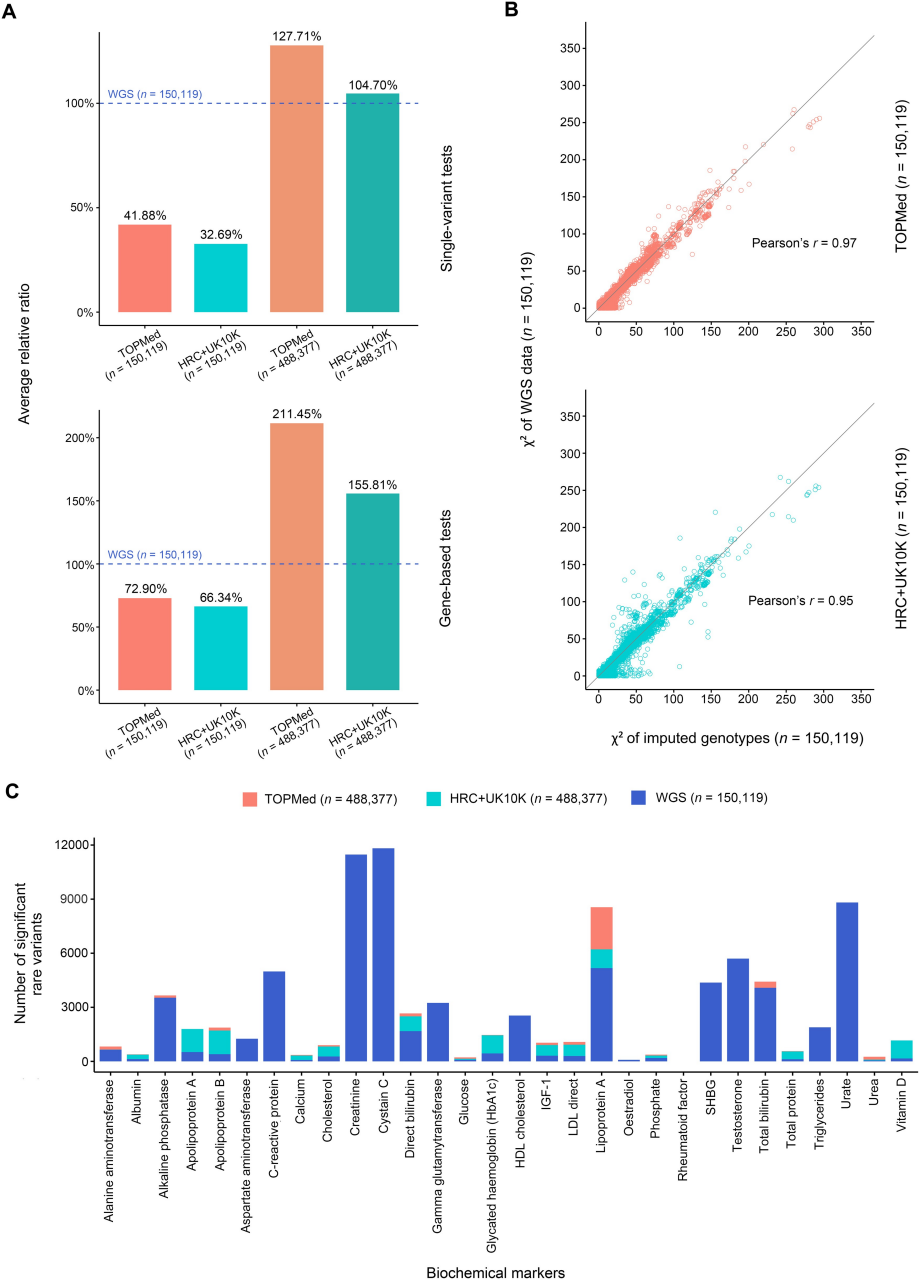
Rare variant association results for biochemical markers **A**. Bar plots showing the average relative ratios of imputed data with different sample sizes compared to WGS data for single-variant and gene-based tests. The blue dashed line represents the level of the ground-truth WGS data (*n* = 150,119), set at 100%. **B**. Pairwise Pearson correlations between chi-square statistics from association tests using imputed data (*n* = 150,119) and WGS data (*n* = 150,119). **C**. Number of additional significant rare variants (MAF *<* 0.01) identified in the imputed data (*n* = 488,377) compared to the WGS data (*n* = 150,119) for 30 biochemical markers.

In the single-variant tests for 30 biochemical markers, the average number of significant rare variants per trait was 2479, 661, and 450 for WGS, TOPMed-imputed, and HRC+UK10K-imputed data, respectively (Table S7). Using the WGS result as the ground truth (set to 100%), the average relative ratio of significant rare variants identified was 41.88% for TOPMed-imputed data and 32.69% for HRC+UK10K-imputed data (**Figure 4**A). Although both imputed datasets identified less significant variants than WGS at the same sample size, TOPMed-imputed data had stronger discovery ability than HRC+UK10K-imputed data. In the gene-based tests for 30 biochemical markers, the average number of significant genes per trait was 7, 5, and 5 for WGS, TOPMed-imputed, and HRC+UK10K-imputed data, respectively (Table S8). The average relative ratio of significant genes identified was 72.90% for TOPMed-imputed data and 66.34% for HRC+UK10K-imputed data, which were still lower than that for WGS data (100%) (Figure 4A).

In addition, we depicted the chi-square statistics of the association analyses for 30 biochemical markers in WGS data and two imputed datasets. The test statistics in both imputed datasets presented a strong relationship with those in WGS data, indicating that their association test statistics are highly correlated (average Pearson’s *r* = 0.97 for TOPMed-imputed data; average Pearson’s *r* = 0.95 for HRC+UK10K-imputed data) (Figure 4B; Table S9). This demonstrates that the rare variant–trait associations derived from imputed genotypes are robust.

Furthermore, using the WGS association results as ground truth, we calculated the false positive rate of significant variants/genes found in 30 biochemical markers for each imputed dataset (Table S10). The false positive rate was defined as the proportion of variants/genes considered significant in the imputed data but non-significant in the WGS data among all significant variants/genes found in the imputed data. For single-variant tests, the median false positive rate of all traits was 0.33 for the TOPMed-imputed data and 0.40 for the HRC+UK10K-imputed data. For gene-based tests, the median false positive rate of all traits was 0.11 for the TOPMed-imputed data and 0.30 for the HRC+UK10K-imputed data.

In the single-variant tests for 15 complex diseases, both imputed datasets identified an average of 2 significant rare variants per disease, which is the same as the WGS data (Table S11). Setting the average relative ratio for the WGS data to 100%, the corresponding value was 93.32% for the TOPMed-imputed data and 87.29% for the HRC+UK10K-imputed data (Figure S3). In the gene-based tests for 15 complex diseases, TOPMed-imputed data identified a total of 5 significant genes across all diseases, while both HRC+UK10K-imputed and WGS data identified 6 significant genes (Table S12).

In summary, the association results from imputed data were closer to the results from WGS data in gene-based tests than in single-variant association tests. When performance was benchmarked against WGS (set to 100%), TOPMed-imputed data outperformed HRC+UK10K-imputed data in rare variant association analyses.

### Rare variant association analysis for quantitative biochemical markers in 488,377 UKB participants

To further explore the extent to which the imputed data can improve the power of rare variant association analysis when the sample size is increased, we performed association analyses for 30 biochemical markers on the White population using two imputed datasets (sample size: *n* = 488,377) and compared their results with those from the WGS data (*n* = 150,119).

In the single-variant tests, both imputed datasets identified more significant rare variants for 16 traits than WGS data (Figure 4C; Table S7). Specifically, compared to WGS (set at 100%), the average relative ratio of significant rare variants identified increased by 4.7% for the HRC+UK10K-imputed data and by 27.71% for the TOPMed-imputed data (Figure 4A). However, WGS data detected more significant rare variants for 12 traits than both imputed datasets, likely due to the large number of variants in the WGS data (Figure 4C; Table S7). In gene-based tests, the TOPMed-imputed data identified an average of 22 significant genes per trait, the HRC+UK10K-imputed data identified 18, while the WGS data identified only 7 (Table S8). Compared to WGS, the average relative ratio of significant genes identified increased by 55.81% for the HRC+UK10K-imputed data and by 111.45% for the TOPMed-imputed data (Figure 4A).

We also conducted an association analysis of 30 biochemical markers on the Asian and African populations using the WGS data (*n* = 150,119) and the two imputed datasets (*n* = 488,377). In the single-variant tests on the Asian population, the TOPMed-imputed data identified an average of seven significant rare variants per trait, the HRC+UK10K-imputed data identified six, whereas the WGS data identified only two (Table S13). Similarly, in the single-variant tests on the African population, the TOPMed-imputed data identified an average of eight significant variants per trait, compared to seven from the HRC+UK10K-imputed data and one from the WGS data (Table S14). The overall trends in the Asian and African populations were consistent with that in the White population in the single-variant tests. However, in gene-based tests of 30 biochemical markers in these two non-White populations, no significant genes were identified from imputed data and WGS data on average (Tables S15 and S16), likely due to the relatively small sample sizes of Asians (*n* = 9835) and Africans (*n* = 8031) within the imputed SNP array data (*n* = 488,377).

### Rare variant association analysis for complex diseases in 488,377 UKB participants

We next conducted rare variant association analyses for 15 common diseases, including 10 chronic diseases and 5 cancers, on the White population using two imputed datasets (*n* = 488,377), and compared their results to those from the WGS data (*n* = 150,119) (Table S17). The quantile-quantile (QQ) plots of all test results for 15 traits are provided in Figures S4–S9.

**Figure 5:**
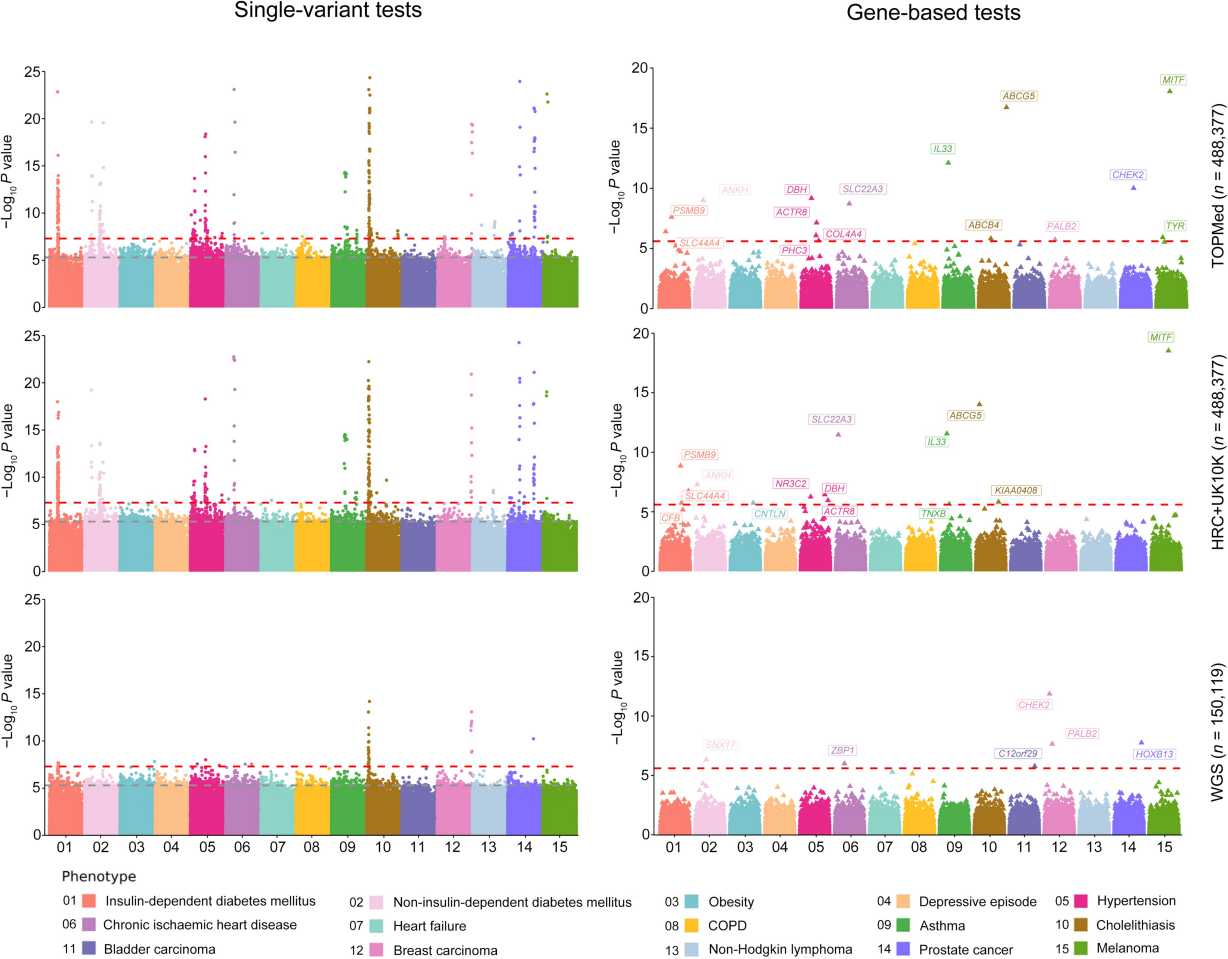
Rare variant association results for complex diseases Multiple-trait Manhattan plots of single-variant tests and gene-based tests for 15 diseases in different datasets. The *x*-axis labels each disease, and the *y*-axis shows −log_10_  *P* value. The red dashed line represents the significance filtering threshold of the *P* value (*P* < 5 × 10^−8^ for single-variant tests; *P* < 2.5 × 10^−6^ for gene-based tests), and the gray dashed line represents the suggestive filtering threshold of the *P* value (*P* < 5 × 10^−6^ for single-variant tests). COPD, chronic obstructive pulmonary disease.

In the single-variant tests, the average relative ratio of significant rare variants identified from the imputed data increased by approximately 10-fold compared to WGS, which was due to the larger sample sizes and sufficient disease cases (Figure S3; Table S18). On average, the TOPMed-imputed data identified 22 significant rare variants, the HRC+UK10K-imputed data identified 24, while the WGS data detected only 2 (**Figure 5**; Table S11). Previous apples-to-apples comparisons indicated that imputed data (*n* = 150,119) had lower rare variant detection capability than WGS (Figure S3). However, when the sample size of the imputed data was increased, it outperformed the smaller WGS dataset and improved the power of rare variant association analysis. These findings suggest that large-scale SNP array-based data imputed using sequencing reference panels may greatly improve rare variant detection compared to WGS data.

In the gene-based tests, the imputed data also identified more significant genes than WGS. The TOPMed-imputed data detected a total of 15 significant genes, the HRC+UK10K-imputed data detected 14, while the WGS data only detected 6 (Figure 5; Tables S12 and S19). In addition, we observed discrepancies in gene identification across imputed datasets and WGS data. For example, some well-established genes were consistently identified in both imputed datasets but were not identified in the WGS data, such as *IL33* (a known asthma-associated gene in which rare mutations can decrease asthma risk) [37,38]. In contrast, certain genes were identified only in specific imputation resources. For instance, *CHEK2*, which has been confirmed as a prostate cancer risk gene harboring disease-associated rare variants [39], was identified in the TOPMed-imputed data and WGS data, but not in the HRC+UK10K-imputed data. Most significant genes identified from the imputed data had supporting associations or biological evidence from previous studies (Table S20). The results above suggest that combining larger SNP array cohorts with imputation using large-scale sequencing reference panels can enhance the ability to detect rare variant–trait associations.

### Powering rare variant discovery by meta-analysis of WGS and SNP array data

**Figure 6:**
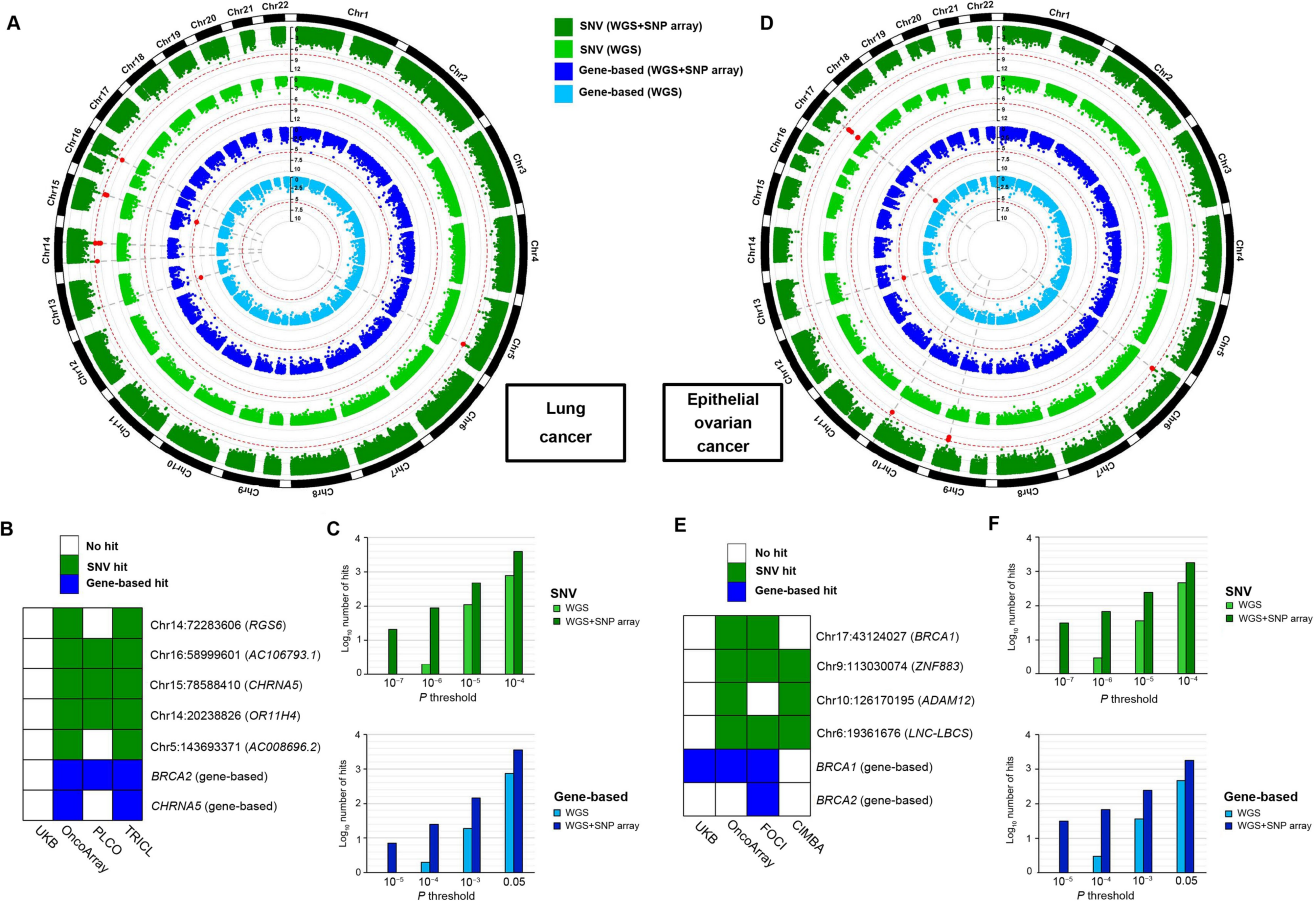
Pooling WGS and SNP array-imputed data for association analyses of lung cancer and epithelial ovarian cancer All SNP array datasets were imputed using the TOPMed reference panel and then subjected to association analyses. **A**. Circos plot showing the single-variant and gene-based association results for lung cancer using UKB WGS data alone or WGS+SNP array data. **B**. Rare variants and genes associated with lung cancer identified in each dataset. The blocks are colored if the *P* values reach nominal significance (*P* < 0.05). **C**. Comparison of the number of identified signals for lung cancer in single-variant and gene-based association tests at different *P* value thresholds. **D**. Circos plot showing the single-variant and gene-based association results for epithelial ovarian cancer using UKB WGS data alone or WGS+SNP array data. **E**. Rare variants and genes associated with epithelial ovarian cancer identified in each dataset. The blocks are colored if the *P* values reach nominal significance (*P* < 0.05). **F**. Comparison of the number of identified signals for epithelial ovarian cancer in single-variant and gene-based association tests at different *P* value thresholds. SNV, single-nucleotide variant.

Furthermore, we conducted rare variant association analyses for LC and EOC using both SNP array and WGS data. For each cancer, we analyzed three SNP array datasets imputed using the TOPMed reference panel, along with the UKB WGS data. Then, meta-analyses were performed for two cancers separately on their association results of the three SNP array datasets and the WGS data (**Figure 6**A and D). Compared to the limited cases in the WGS data (*n*_LC_ = 1494; *n*_EOC_ = 628), the SNP array data provided sufficient cases (*n*_LC_ = 23,168; *n*_EOC_ = 23,822), representing 16-fold and 38-fold for LC and EOC, respectively.

In the single-variant tests, no rare variants passed the genome-wide significance threshold in WGS results. However, when combining with the association results of SNP array data and WGS data using METAL [40], we detected 12 and 22 significant SNVs for LC and EOC, mapping to 5 and 4 independent genetic regions, respectively (Figure 6B and E; Tables S21 and S22). In the gene-based tests, only *BRCA1* in EOC passed *P* < 2.5 × 10^−6^ in WGS results. However, meta-analyses identified that *BRCA2* and *CHRNA5* were significantly associated with LC, and that *BRCA2* was also significantly associated with EOC (Figure 6B and E; Tables S23 and S24). Across a range of *P* value thresholds, the meta-analysis strategy for WGS+SNP array detected more variants, especially under stringent thresholds (*e.g.*, 10^−7^ for single-variant tests and 10^−5^ for gene-based tests) (Figure 6C and F). The QQ plots of meta-analysis results for the two cancers showed no statistical inflation (Figure S10).

## Discussion

Large-scale biomarker-based prospective cohorts are emerging, especially focusing on population genomics [41]. However, limited by the cost of NGS technology, biobank-level sequencing data remain in the minority, making it difficult to explore rare variants that provide important genomic architecture and heritability explanation. While large-scale GWASs primarily leverage imputed genotype data of common variants, there exists a notable lack of systematic comparison of the power of imputed data for rare variants. Therefore, evaluating the consistency between rare variants identified from SNP array data imputed using two frequently used large-scale sequencing reference panels and those identified from WGS data, along with quantifying the power of imputed data in rare variant association analysis, can provide better guidance and reference for using imputed data to explore the associations between rare variants and complex traits when population WGS data are unavailable.

Genotype imputation from a large sequencing population requires substantial hardware and computational resources, and using TOPMed or HRC reference panel is currently the easiest approach to implement due to their convenient online imputation servers [42]. Considering this situation, we conducted a comprehensive series of analyses of rare variant imputation using SNP array data from UKB, based on TOPMed or HRC+UK10K, with the WGS data of 150,119 individuals as the ground truth. We described the rare variant imputation results, and carried out genotype consistency analyses in three ethnic groups (White, Asian, and African) from the UKB (matched to samples of WGS data) to evaluate the accuracy of rare variant imputation. We further performed rare variant association analyses under two sample size scenarios (*n* = 150,119 and *n* = 488,377) to assess the performance of imputed rare variants at both single-variant and gene-based levels.

Our results showed that the TOPMed panel could impute more high-quality rare variants than the HRC+UK10K panel. Although the imputed variants were fewer than WGS in singleton and doubleton variants, the remaining rare variants imputed by TOPMed had a certain scale. Even for extremely rare variants (MAC ≤ 5), the average imputation quality of TOPMed-imputed variants was acceptable (INFO/RSQ > 0.6). Although not every rare variant achieved a high imputation quality, the overall imputation quality of rare variants was still considerable, especially for the TOPMed-imputed data.

For the three ethnic groups, we observed that rare variants imputed using TOPMed were closer to WGS data than those imputed using HRC+UK10K in each MAC interval. For Whites, the consistency of rare variants with MAC ≤20 in HRC+UK10K-imputed data was poor, which was not recommended for subsequent application. In addition, the overall correlation between TOPMed-imputed and WGS data was slightly stronger in Africans than in the other two ethnic groups, which may be due to the small sample size of Africans (*n* < 1000) in the WGS data. Since Cramer’s V and Pearson’s *r*^2^ can both be affected by the sample size, it is not appropriate to directly compare the coefficients under unbalanced ethnic sample sizes. Other factors affecting the differences in correlation metrics between different ethnic groups need further study. Nevertheless, the stable and robust performance of TOPMed-imputed data supports its reliability for rare variant association studies. On the whole, TOPMed-imputed data performed better in correlation analyses with WGS data than HRC+UK10K-imputed data, in line with expectations based on their panel composition. The TOPMed reference panel comprises 97,256 participants with ethnic and ancestral diversity. It contains 410,323,831 genetic variants, of which 78.7% have not previously been described. Furthermore, over 97% of the variants in TOPMed are rare variants [24]. By contrast, the HRC reference panel comprises 32,488 participants of predominantly European ancestry, containing 39,741,659 variants (representing approximately one-tenth the variant count of TOPMed). A study has demonstrated that for imputing rare variants (MAF < 0.005) in admixed African and Hispanic/Latino populations, the TOPMed panel outperforms the HRC and 1000 Genomes Project [43].

In the association analysis of quantitative and binary traits among White individuals from the 150,119 UKB participants, although the ability of both imputed datasets to identify significant rare variants was weaker than that of WGS data, we could still identify a few significant rare variants or genes. In the scenario of the same sample size as WGS, the false positive rate of significant variants/genes found by TOPMed-imputed data was significantly lower than that of HRC+UK10K-imputed data. These results suggest that, if WGS data are lacking, it is more reliable to use TOPMed-imputed data for rare variant association analysis. However, we also observed that for certain traits, particularly those influenced by predominantly ultra-rare variants, such as creatinine, the false positive rate remained markedly high even using TOPMed-imputed data. This likely reflects the difficulty of accurately imputing ultra-rare variants, which may lead to failure in capturing true associations in some cases. While imputation-based approaches remain valuable and practical alternatives to WGS, especially when large-scale sequencing data are not available, they are generally more reliable for low-frequency variants than for ultra-rare ones.

When the sample size of the imputed data increased to 488,377, the ability to identify significant rare variants improved, but this improvement varied slightly in distinct types of traits. For quantitative traits, the power of WGS data was basically sufficient, and the two imputed datasets had limited improvement. However, for binary traits, the number of disease cases in WGS data was far from sufficient to ensure optimistic power, leading to fewer significant rare variants detected. This challenge is compounded by the well-documented case-control imbalance problem prevalent in natural population cohorts, where adequate sample sizes are often accompanied by insufficient disease cases, ultimately resulting in low statistical power to identify rare variants [44]. To improve the power, external data with additional disease cases should be supplemented. Our study indeed showed that the larger SNP array dataset imputed using large-scale sequencing reference panels, particularly the TOPMed reference panel, greatly improved the ability to find significant rare variants across all three ethnic groups. Also, we performed meta-analyses on the association analysis results of WGS data and multiple SNP array datasets for CL and EOC, respectively. We successfully identified classical rare variants and genes associated with two cancers, including well-known *BRCA2* [45], *BRCA1* [46], and *CHRNA5* [47], which could not be detected from the UKB WGS data alone. These findings demonstrate that, under certain conditions, imputed data can achieve analytical performance comparable to WGS data, thereby serving as a cost-effective alternative for rare variant association analyses. Nonetheless, we also found that even when the total number of significant genes identified was similar across WGS and imputed datasets, the overlap of specific genes was often limited. Certain genes were uniquely identified in a single dataset, suggesting that rare variant signals are sometimes dataset-specific, possibly due to imputation uncertainty, allele frequency differences, or trait-specific genetic architecture. Further studies are needed to clarify the exact reasons for this limited overlap.

There are several strengths in our study. First, we comprehensively described the landscape of imputed rare variants using different sequencing panels in terms of variant amount, coverage, and imputed quality, and compared them with the WGS data. Second, we leveraged WGS data as the ground truth to perform correlation analyses by different ethnic groups and analyzed the consistency of imputed rare variants with corresponding sequencing variants. We demonstrated the feasibility of using imputed rare variants for the association analysis. Third, we set up two sample size scenarios (one with 150,119 individuals matching the WGS data and the other with the sample size increased to 488,377) to conduct various association tests on 45 traits of the UKB and to evaluate analysis power. Fourth, we collected multiple case-control SNP array GWASs for the two cancers and performed meta-analyses with the WGS association results, illustrating the supplementary value of the imputed SNP array data to WGS.

The results we presented here also have some limitations. First, our genotype consistency evaluation was conducted only on the UKB SNP array (∼  50,000 UK BiLEVE Axiom array and ∼  450,000 UKB Axiom array) that did not consider other array types, which might influence the results. Second, we performed genotype imputation on the TOPMed’s online server (97,256 reference samples) using Minimac4 [42], which may lead to slight differences compared with results obtained using other imputation software. However, TOPMed’s imputation online server is convenient and feasible for most genomic studies. Theoretically, the results of imputation in larger populations (*e.g.*, UKB whole WGS population) should be more accurate [32], which needs to be confirmed. Third, we attempted to integrate the SNP array data and WGS data in two exemplary cancers. The effect of integrating other diverse diseases in large-scale population cohorts (*n*  ≥ 100,000) needs further evaluation.

In conclusion, in large-scale rare variant association studies, when WGS data of the corresponding population are insufficient to acquire, imputation based on the TOPMed reference panel can be selected as a relatively reliable approach, while its false positive risk needs to be reasonably considered. The imputed data with large sample sizes can enhance the analytical power beyond WGS data with small sample sizes in rare variant association studies. Our study also suggests that the association results from SNP array case-control data with sufficient cases, when combined with WGS data, can enhance the power of WGS-only analysis to identify significant rare variants/genes. Therefore, this integrative strategy deepens our understanding of how rare genetic variants contribute to human complex traits and diseases.

## Materials and methods

### WGS data collection and variant calling

We collected WGS data of 150,119 individuals from the UKB (data field 23352) [48], which were sequenced to an average coverage of 32.5× (minimum 23.5× per individual) on Illumina NovaSeq sequencing platform at deCODE Genetics (90,667 individuals) and the Wellcome Trust Sanger Institute (59,452 individuals). Sequence reads were mapped to the human reference genome GRCh38 using Burrows–Wheeler Alignment (BWA), an efficient read alignment tool based on Burrows–Wheeler Transform [49]. SNPs and short insertions/deletions (indels) were jointly called over all individuals using GraphTyper (v2.7.1) [50], which is a fast and highly scalable program for both small and population-scale sequencing studies and provides more accurate genotype calls. This yielded a set of high-quality variants, including 585,040,410 SNVs and 58,707,036 indels.

### Genotype imputation of SNP array data using HRC+UK10K and TOPMed reference panels

SNP array data were collected from 488,377 UKB individuals (including 150,119 with matched WGS data). To infer genotypes not directly detected by the SNP array platform due to its technical limitations (Figure S1), we performed imputation using two mainstream reference panels: the HRC+UK10K panel (the HRC panel supplemented by the UK10K+1000 Genomes panel) and the TOPMed R2 panel.

The HRC+UK10K panel increases the number of testable variants by over 100-fold, yielding approximately 96 million variants (data field 22828). In this approach, the HRC panel (64,976 haplotypes) was used wherever possible. For variants absent from the HRC panel, the UK10K+1000 Genomes panel (12,570 haplotypes) was used instead [51]. The raw genomic positions were then lifted over from GRCh37 to GRCh38 coordinates. Phasing of 670,739 autosomal markers was carried out using SHAPEIT3 [52] in chunks of 15,000 markers with a 250-marker overlap. Imputation was then performed using IMPUTE4 [53] — a faster and more memory-efficient haploid imputation tool than IMPUTE2 [51] — in chunks of approximately 50,000 imputed markers with a 250-kb buffer region and on 5000 samples per compute job.

For the second approach, imputation was performed using the TOPMed R2 panel (97,256 deeply sequenced genomes) from the TOPMed Informatics Research Center [24]. Across all chromosomes, 677,037 markers were used for imputation. The UKB genetic data were first phased in 81 chromosomal chunks using Eagle (v.2.4) [54], which improves accuracy over previous methods. The phased data were then converted from GRCh37 to GRCh38 using LiftOver [55]. Imputation was performed using Minimac4 (v1.0.2) [42], which is appropriate for low-frequency markers and utilizes less imputation tools’ memory [31]. Imputation was performed in 1-Mb chunks, which were subsequently merged to generate chromosome-wide imputed data.

### Quality control of imputed SNP array data

Prior to GWAS analyses using the SNP array data of 488,377 UKB individuals, we performed quality control on the imputed genotypes. Variants with an imputation quality greater than 0.3 [MACH $R^{2}$ (RSQ) > 0.3 or INFO > 0.3] were retained, as this threshold can provide association power comparable to that obtained from the perfect genotypes in the 150,119 WGS samples. The two imputation quality metrics, RSQ and INFO, were calculated by Minimac4 and IMPUTE4, respectively [42,56–58].

Let H represent a set of N haplotypes of L SNPs, and let G represent the genotype data of K individuals with these L SNPs. Thus, $G_{ij}\in \{0,1,2\}\;(i=1,\ldots ,K;j=1,\ldots ,L)$ represents the genotype at the j-th SNP of the i-th individual, and $p_{\mathrm{ijk}}=P(G_{ij}=k|H,G)$ denotes the probabilistic prediction generated by imputation. This implies that the expected allele dosage for the genotype at the j-th SNP of the i-th individual is $e_{ij}=p_{ij1}+2p_{ij2}$ and $f_{ij}=p_{ij1}+4p_{ij2}$. Let θ represent the population allele frequency, so that estimate $\hat{\theta }=\frac{\sum_{i=1}^{N}e_{ij}}{2N}$. The MACH $R^{2}$ (RSQ) at the j-th SNP is defined as:


(1) $$R^{2}=\{\begin{array}{cc} \frac{\frac{\sum_{i=1}^{N}e_{ij}^{2}}{N}-{\left(\frac{\sum_{i=1}^{N}e_{ij}}{N}\right)}^{2}}{2\hat{\theta }(1-\hat{\theta })}, & \mathrm{when}\;\hat{\theta }\in (0,1)\\ 1, & w\mathrm{hen}\;\hat{\theta }=0,\hat{\;\theta }=1 \end{array}$$

The more accurate the imputed genotypes are, the closer MACH $R^{2}$ (RSQ) approaches to 1, although it may exceed 1.

The INFO score at the *j*-th SNP is defined as:


(2) $$\mathrm{INFO}\;\mathrm{score}=\{\begin{array}{cc} 1-\frac{\sum_{i=1}^{N}\left(f_{ij-e_{ij}^{2}}\right)}{2N\hat{\theta }(1-\hat{\theta })}, & \mathrm{when}\;\hat{\theta }\in (0,1)\\ 1, & w\mathrm{hen}\;\hat{\theta }=0,\;\hat{\theta }=1 \end{array}$$

Similarly, when the genotypes are imputed with high certainty, the INFO score approaches 1, with 1 being its upper bound [57]. Both metrics represent the expected imputation quality for each variant.

### Genotype consistency evaluation

In the WGS and two imputed (TOPMed and HRC+UK10K) datasets, we stratified the individuals into three ethnic groups — White, Asian, and African — based on self-reported ethnic background (data field 21000). To evaluate the consistency between the imputed and WGS genotypes, we matched individuals in the imputed data (*n* = 488,377) to those in the WGS data (*n* = 150,119) within each ethnic group using participant IDs. Then, we used PLINK (v2.00a3LM) to calculate MAC and MAF of each genetic variant in each ethnic group. Variants observed with a minor allele only once in the entire sample were defined as singletons (MAC = 1), and those observed twice as doubletons (MAC = 2). Rare variants were defined as those with MAF < 0.01, and ultra-rare variants as those with MAF < 0.0001 (excluding singletons and doubletons). Meanwhile, based on MAC and MAF, genetic variants were classified into five categories: MAC∈(0, 5], (5, 10], (10, 20], (20, 50], and MAC > 50 (abbreviated as > 50 in subsequent analyses), along with MAF < 0.01. We chose Cramer’s V as the primary metric to evaluate the consistency between imputed variants and WGS variants:


(3) Cramer’s V=χ2nm

where χ^2^ represents the chi-square statistic from a 3×3 contingency table consisting of the genotype counts (genotype  ∈{0,1,2}) from the WGS and imputed (TOMed or HRC+UK10K) data; *n* represents the sample size; and *m* represents the smaller value of the two degrees of freedom (*r−*1) or (*c−*1) of the two variables, where *r* is the number of rows and *c* is the number of columns in the contingency table. Cramer’s V is a correlation coefficient with a value lie in the range [0, 1]. The closer its value is to 1, the stronger the correlation between row and column variables. Since our study focuses on rare variants, an average Cramer’s V > 0.7 is considered a good performance. Besides, we computed another common metric, Pearson’s squared correlation (Pearson’s *r*^2^), as an additional measure of genotype consistency analyses.

### Single-variant and gene-based association tests

Association analyses were performed for 45 traits (30 biochemical markers and 15 complex diseases). All phenotype data were accessed in July 2022 and processed on DNAnexus Research Analysis Platform (RAP). Blood biochemical data were collected from UKB Category 17518, which includes the measurements from a project that quantified a broad panel of biochemical markers in biological samples collected at baseline (2006–2010) of all 500,000 participants. All 30 biochemical markers were analyzed as quantitative traits. Health-related outcomes were ascertained by linking participant records to national cancer registries, mortality registries, and hospital inpatient encounters. Diagnoses in these sources were coded by International Classification of Diseases version 10 (ICD-10). Corresponding ICD-10 codes within the UKB were extracted from data fields 41270 (Diagnoses-ICD10), 41202 (Diagnoses-main ICD10), and 40001 (primary cause of death: ICD10). Individuals with at least one recorded incident diagnosis were defined as cases. We included 15 common chronic diseases or cancers as binary traits: insulin-dependent diabetes mellitus, non-insulin-dependent diabetes mellitus, obesity, depressive episode, hypertension, chronic ischemic heart disease, heart failure, chronic obstructive pulmonary disease, asthma, cholelithiasis, bladder carcinoma, breast carcinoma, non-Hodgkin lymphoma, prostate cancer, and melanoma.

Single-variant and gene-based association analyses were performed using REGENIE (v3.2.6) [59], a machine learning-based method for fitting whole-genome regression models for quantitative and binary phenotypes. Quantitative traits were inverse-normal transformed based on rank. Saddlepoint approximation (SPA) was used to address extreme case-control imbalance in binary traits [60]. Association tests were performed separately in three ethnic groups.

The single-variant association tests included rare variants with MAF < 0.01. Variants were functionally annotated using *V*ariant Effect Predictor (VEP) [61]. The genome-wide significance threshold of single-variant tests was set to *P* < 5 × 10^−8^.

For gene-based analyses, we also included rare variants with MAF < 0.01. Three genetic models were considered: LoF, LoF+missense, and LoF+missense+synonymous. Among all combinations, the association results with the smallest *P* value were reported to collectively capture a wide range of genetic architectures [5]. The genome-wide significance threshold of gene-based tests was set to *P* < 2.5 × 10^−6^.

In the association analyses of quantitative traits, we adjusted the covariates including age, sex, and the first ten principal components (PCs). In the association analyses of binary traits, we adjusted the covariates including age, sex (excluding sex-specific diseases), body mass index (BMI), smoking status (binary), drinking status (binary), and the first ten PCs.

Additionally, we defined a relative ratio for each trait. We denoted the difference between the number of significant variants/genes identified in the imputed data and that in the WGS data as *diff* ($\mathrm{diff}={\mathrm{number}}_{\mathrm{imputed}}-{\mathrm{number}}_{\mathrm{WGS}}$, considering only *diff*  > 0), and the number of significant variants/genes identified in the WGS data as the standard number, *std*. Then the relative ratio was calculated as *diff* / *std*. The average relative ratio was obtained by averaging the ratios across all traits in the same type (quantitative or binary). Considering WGS as the ground truth, the average relative ratio for WGS data was set to 100%, which could be the comparison standard for evaluating imputed data.

### SNP array data collection and meta-analyses for LC and EOC

For LC, three SNP array datasets were collected from the Prostate, Lung, Colorectal, and Ovarian (PLCO) cancer screening trial [62], the International Lung Cancer OncoArray Consortium (ILCCO-OncoArray) [63], and the Transdisciplinary Research in Cancer of the Lung (TRICL) research team [45] (Table S25). For EOC, three SNP array datasets were collected from the Follow-up of Ovarian Cancer Genetic Association and Interaction Studies (FOCI)-OncoArray [64], the FOCI-Exome Chip [65], and the Consortium of Investigators of Modifiers of BRCA1/2 (CIMBA) [66] (Table S26). Samples were excluded if they lacked disease status, were second-degree relatives or closer [identity by descent (IBD) > 0.2], had low-quality DNA (call rate < 95%), had sex inconsistency, or were of non-European ancestry. SNPs were removed if they met any of the following criteria: (1) located on sex chromosomes, (2) MAF < 0.05, (3) call rate < 95%, and (4) Hardy–Weinberg equilibrium (HWE) test *P* < 1.00 × 10^−7^ in controls or *P* < 1.00 × 10^−12^ in cases. All genotype data were imputed on the TOPMed online imputation server. SNVs with poor imputation quality (INFO/RSQ < 0.3) and located on sex chromosomes were excluded. Following these steps, rare variant association analyses for LC and EOC were conducted using both SNP array-imputed and WGS data.

Fixed-effect meta-analyses were then performed to summarize the single-variant association results from the WGS and three SNP array-imputed datasets using METAL [40]. The effect sizes and 95% confidence interval (CI) of genes were estimated using burden tests and then summarized by fixed effect meta-analysis.

## Ethical statement

The UKB is a population-based prospective cohort of individuals aged 40–69 years, enrolled between 2006 and 2010 [67]. The work described herein was approved by the UKB (Application Nos. 92675 and 83445).

## Code availability

The analysis code used in this study is available at GitHub (https://github.com/djl356/Imputation_Project). The code has also been submitted to BioCode at the National Genomics Data Center (NGDC), China National Center for Bioinformation (CNCB) (BioCode: BT007792), which is publicly accessible at https://ngdc.cncb.ac.cn/biocode/tool/BT007792.

## CRediT author statement

**Jinglan Dai:** Visualization, Methodology, Software, Investigation, Formal analysis, Writing – original draft, Writing – review & editing. **Yixin Zhang:** Investigation, Data curation, Writing – review & editing. **Yuan Gao:** Formal analysis, Data curation, Writing – review & editing. **Hongru Li:** Investigation, Supervision. **Sha Du:** Investigation, Supervision. **Hao Hong:** Investigation, Supervision. **Dongfang You:** Investigation, Supervision. **Zaiming Li:** Investigation, Supervision. **Ruyang Zhang:** Investigation, Supervision. **Yang Zhao:** Investigation, Supervision, Funding acquisition, Resources. **Zhonghua Liu:** Investigation, Supervision. **David C. Christiani:** Investigation, Supervision, Funding acquisition, Resources. **Feng Chen:** Conceptualization, Methodology, Funding acquisition, Resources, Supervision. **Sipeng Shen:** Visualization, Conceptualization, Methodology, Software, Funding acquisition, Resources, Supervision, Writing – original draft, Writing – review & editing. All authors have read and approved the final manuscript.

## Competing interests

The authors have declared no competing interests.

## Supplementary Material

qzaf084_Supplementary_Data

## ORCID

ORCID 0009-0005-0944-9757 (Jinglan Dai)

ORCID 0009-0005-8280-2422 (Yixin Zhang)

ORCID 0009-0005-7523-4551 (Yuan Gao)

ORCID 0009-0005-1527-4500 (Hongru Li)

ORCID 0009-0005-5023-7369 (Sha Du)

ORCID 0009-0000-3114-7767 (Hao Hong)

ORCID 0000-0002-0856-0323 (Dongfang You)

ORCID 0009-0007-0366-3095 (Zaiming Li)

ORCID 0000-0003-3861-4297 (Ruyang Zhang)

ORCID 0000-0003-1393-7567 (Yang Zhao)

ORCID 0000-0003-3048-9823 (Zhonghua Liu)

ORCID 0000-0002-0301-0242 (David C. Christiani)

ORCID 0000-0002-2699-7190 (Feng Chen)

ORCID 0000-0003-0436-4736 (Sipeng Shen)
