# Supplementary material for: Boosting the Power of Rare Variant Association Studies by Imputation Using Large-scale Sequencing Population
Source: Genomics Proteomics Bioinformatics. 2025 Sep 17;23(5):qzaf084. doi: 10.1093/gpbjnl/qzaf084 (PMC13005946; doi:10.1093/gpbjnl/qzaf084)
Supplement: qzaf084_Supplementary_Data [file qzaf084_supplementary_data.zip › Figure S1.pdf]

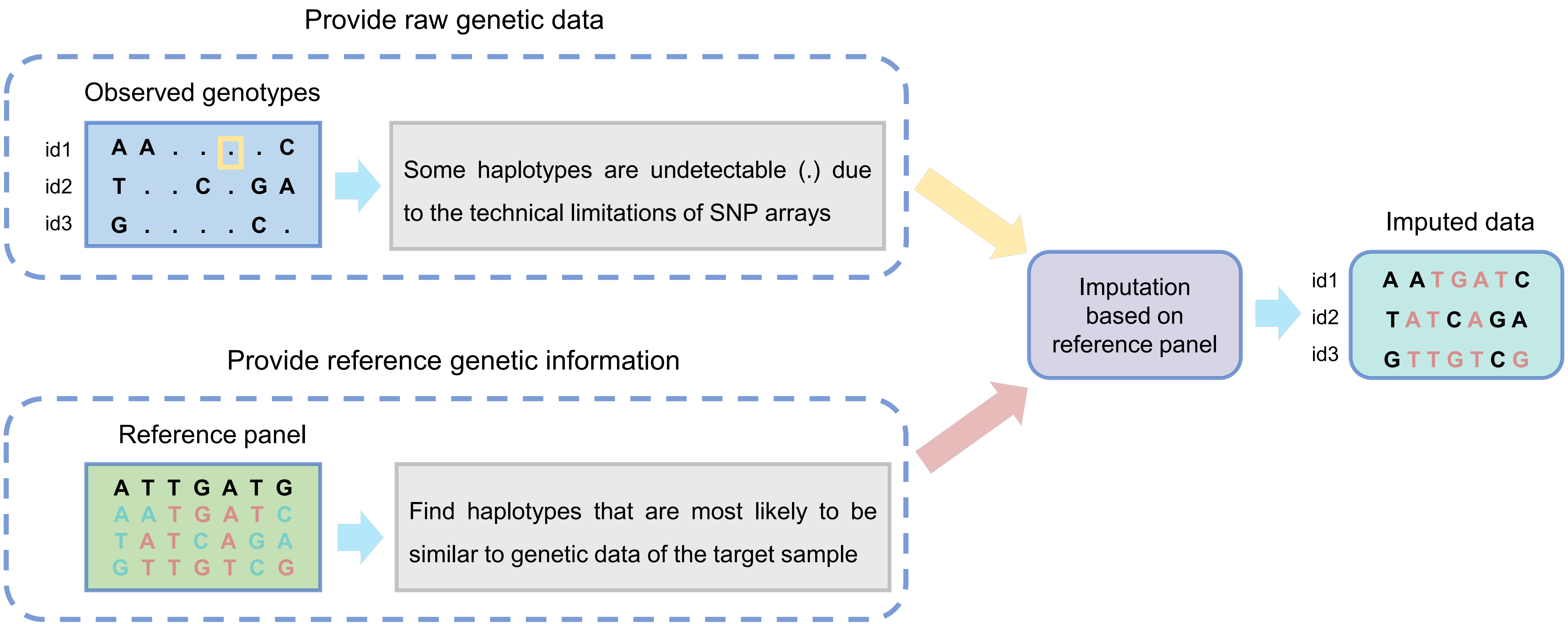

Provide raw genetic data
Observed genotypes
id1
id2
id3
A A C
T . . C . G A
G . . . . C .
Some haplotypes are undetectable (.) due to the technical limitations of SNP arrays
Provide reference genetic information
Reference panel
A T T G A T G
A A T G A T C
T A T C A G A
G T T G T C G
Find haplotypes that are most likely to be similar to genetic data of the target sample
Imputation based on reference panel
Imputed data
id1
id2
id3
A A T G A T C
T A T C A G A
G T T G T C G
