# Supplementary material for: Boosting the Power of Rare Variant Association Studies by Imputation Using Large-scale Sequencing Population
Source: Genomics Proteomics Bioinformatics. 2025 Sep 17;23(5):qzaf084. doi: 10.1093/gpbjnl/qzaf084 (PMC13005946; doi:10.1093/gpbjnl/qzaf084)
Supplement: qzaf084_Supplementary_Data [file qzaf084_supplementary_data.zip › Figure S5.pdf]

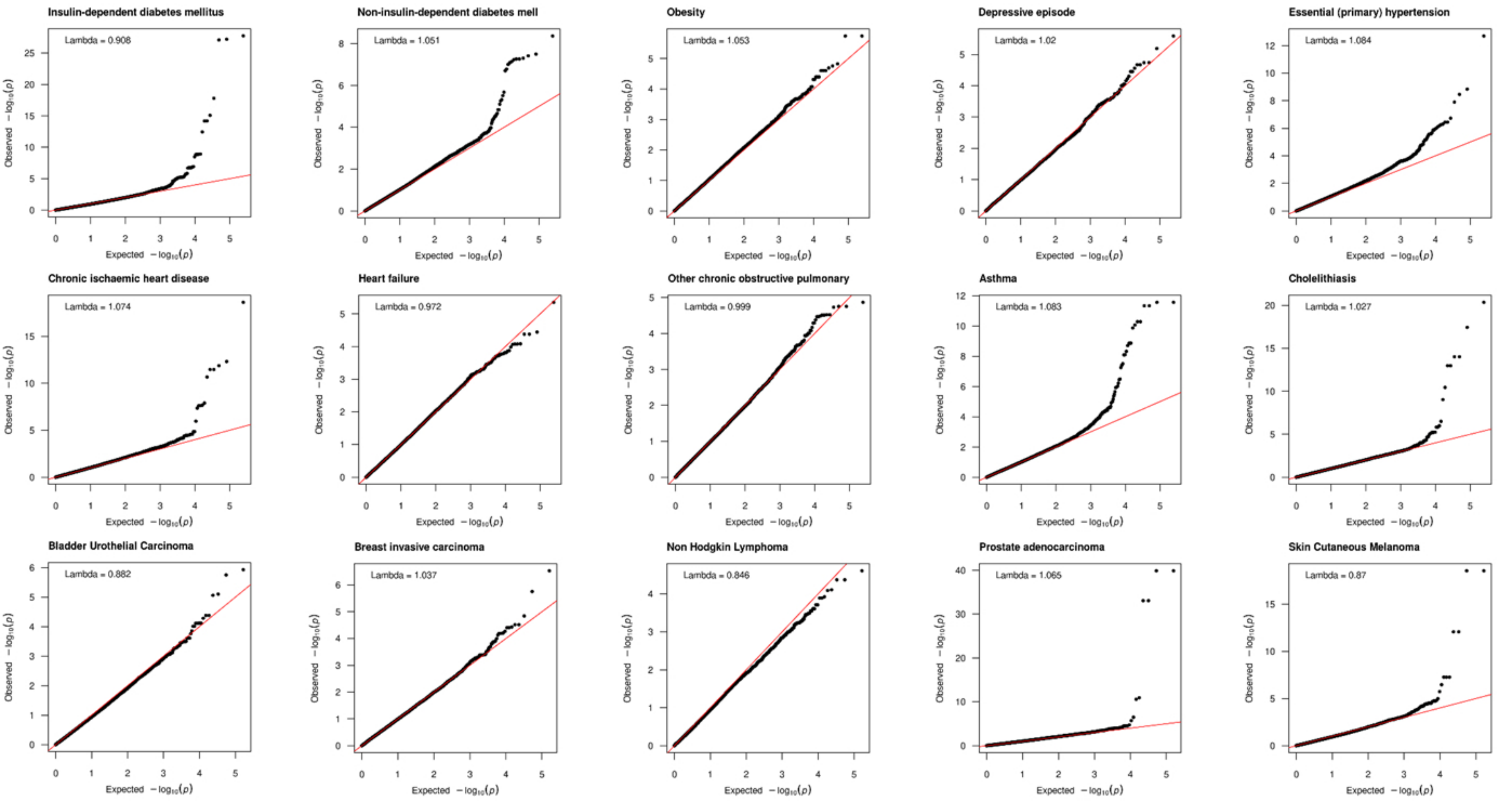
Insulin-dependent diabetes mellitus
Lambda = 0.908
Non-insulin-dependent diabetes mell
Lambda = 1.051
Obesity
Lambda = 1.053
Depressive episode
Lambda = 1.02
Essential (primary) hypertension
Lambda = 1.084
Chronic ischaemic heart disease
Lambda = 1.074
Heart failure
Lambda = 0.972
Other chronic obstructive pulmonary
Lambda = 0.999
Asthma
Lambda = 1.083
Cholelithiasis
Lambda = 1.027
Bladder Urothelial Carcinoma
Lambda = 0.882
Breast invasive carcinoma
Lambda = 1.037
Non Hodgkin Lymphoma
Lambda = 0.846
Prostate adenocarcinoma
Lambda = 1.065
Skin Cutaneous Melanoma
Lambda = 0.87
Observed $-\log_{10}(p)$
Expected $-\log_{10}(p)$
