# Supplementary material for: Boosting the Power of Rare Variant Association Studies by Imputation Using Large-scale Sequencing Population
Source: Genomics Proteomics Bioinformatics. 2025 Sep 17;23(5):qzaf084. doi: 10.1093/gpbjnl/qzaf084 (PMC13005946; doi:10.1093/gpbjnl/qzaf084)
Supplement: qzaf084_Supplementary_Data [file qzaf084_supplementary_data.zip › Figure S7.pdf]

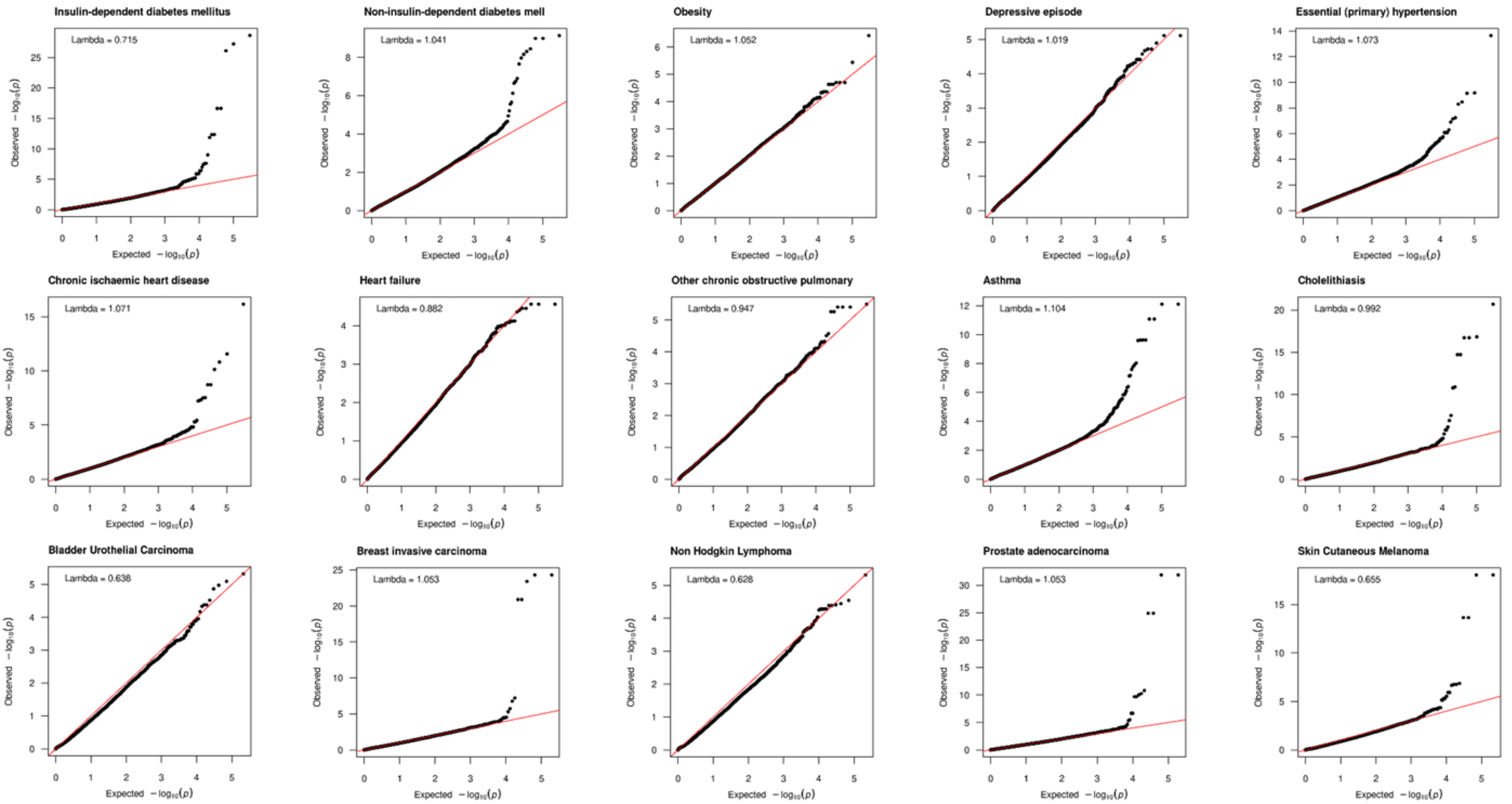
Insulin-dependent diabetes mellitus
Lambda = 0.715
Non-insulin-dependent diabetes mell
Lambda = 1.041
Obesity
Lambda = 1.052
Depressive episode
Lambda = 1.019
Essential (primary) hypertension
Lambda = 1.073
Chronic ischaemic heart disease
Lambda = 1.071
Heart failure
Lambda = 0.882
Other chronic obstructive pulmonary
Lambda = 0.947
Asthma
Lambda = 1.104
Cholelithiasis
Lambda = 0.992
Bladder Urothelial Carcinoma
Lambda = 0.638
Breast invasive carcinoma
Lambda = 1.053
Non Hodgkin Lymphoma
Lambda = 0.628
Prostate adenocarcinoma
Lambda = 1.053
Skin Cutaneous Melanoma
Lambda = 0.655
Observed $-\log_{10}(p)$
Expected $-\log_{10}(p)$
