# Supplementary material for: Boosting the Power of Rare Variant Association Studies by Imputation Using Large-scale Sequencing Population
Source: Genomics Proteomics Bioinformatics. 2025 Sep 17;23(5):qzaf084. doi: 10.1093/gpbjnl/qzaf084 (PMC13005946; doi:10.1093/gpbjnl/qzaf084)
Supplement: qzaf084_Supplementary_Data [file qzaf084_supplementary_data.zip › Supplementary material captions.docx]

# Supplementary material

**Figure S1 Schematic diagram of the imputation mechanism for missing genotypes**

Genotype imputation on the raw genetic data refers to the impute genotypes that actually exist but cannot be detected due to technical limitations of SNP arrays

**Figure S2** **Pearson’s squared correlation (Pearson’s** $\boldsymbol{r}^{\boldsymbol{2}}$**) of three ethnicities between imputed data and WGS data in different MAC intervals**

**Figure S3 Average** **relative ratio in finding significant associations in complex disease association tests**

The x-axis represents different datasets, and the y-axis represents the average relative ratio for all 15 traits, where WGS is the gold standard and its ratio is set to 100%.

**Figure S4 QQ-plots of single-variant tests results for complex diseases for HRC+UK10K-imputed data**

Complex diseases include 10 common diseases (the first two lines) and 5 cancers (the last line).

**Figure S5 QQ-plots of gene-based tests results for complex diseases for HRC+UK10K-imputed data**

Complex diseases include 10 common diseases (the first two lines) and 5 cancers (the last line).

**Figure S6 QQ-plots of single-variant tests results for complex diseases for TOPMed-imputed data**

Complex diseases include 10 common diseases (the first two lines) and 5 cancers (the last line).

**Figure S7 QQ-plots of gene-based tests results for complex diseases for TOPMed-imputed data**

Complex diseases include 10 common diseases (the first two lines) and 5 cancers (the last line).

**Figure S8 QQ-plots of single-variant tests results for complex diseases for WGS data**

Complex diseases include 10 common diseases (the first two lines) and 5 cancers (the last line).

**Figure S9 QQ-plots of gene-based tests results of for complex diseases for WGS data**

Complex diseases include 10 common diseases (the first two lines) and 5 cancers (the last line).

**Figure S10 QQ-plots of meta-analysis results of association analysis results for three SNP array data and WGS data**

Left plot is for lung cancer and right plot is for epithelial ovarian cancer.

**Table S1 Numbers of SNVs in imputed data and WGS data**

**Table S2 The average of the INFO/RSQ**

**Table S3 The variant coverage of different imputed datasets**

**Table S4 Overview of the composition of different ethnic groups in different datasets**

**Table S5 The measures of correlation analyses in different ethnic groups**

**Table S6 The average Cramer's v of two imputed datasets and its difference in three ethnic groups**

**Table S7 (White) The number of significant rare variants with *P* < 5×10^-8^ in single-variant tests for 30 biochemistry markers**

**Table S8 (White) The number of significant genes with *P* < 2.5×10^-6^ in gene-based tests for 30 biochemistry markers**

**Table S9 The Pearson' r between the chi-square statistics of imputed data and of WGS data in association tests for 30 biochemistry markers**

**Table S10 The false positive rate for imputed data (*n* = 150,119) association analysis results of 30 biochemistry markers**

**Table S11 (White) The number of significant rare variants with *P* < 5×10^-8^ in single-variant tests for 15 diseases**

**Table S12 (White) The number of significant genes with *P* < 2.5×10^-6^ in gene-based tests for 15 diseases**

**Table S13 (Asian) The number of significant rare variants with *P* < 5×10^-8^ in single-variant tests for 30 biochemistry markers**

**Table S14 (Arfican) The number of significant rare variants with *P* < 5×10^-8^ in single-variant tests for 30 biochemistry markers**

**Table S15 (Asian) The number of significant genes with *P* < 2.5×10^-6^ in gene-based tests for 30 biochemistry markers**

**Table S16 (African) The number of significant genes with *P* < 2.5×10^-6^ in gene-based tests for 30 biochemistry markers**

**Table S17 (White) Demographic characteristics of the WGS data and UKB GWAS SNP-array data**

**Table S18 (White) Association results of single-variant tests with *P* < 5×10^-8^ for 15 diseases in the imputed data (*n* = 488,377) and WGS (*n* = 150,119)**

**Table S19 (White) Association results for gene-based tests with *P* < 2.5×10^-6^ of 15 diseases in the imputed datasets (*n* = 488,377) and WGS data (*n* = 150,119)**

**Table S20 References list that can support genes found by TOPMed-imputed data (*n* = 488,377)**

**Table S21 Single-variant tests of WGS and SNP-array data in lung cancer**

**Table S22 Single-variant tests of WGS and SNP-array data in epithelial ovarian cancer**

**Table S23 Gene-based tests of WGS and SNP-array data in lung cancer**

**Table S24 Gene-based tests of WGS and SNP-array data in epithelial ovarian cancer**

**Table S25 Demographic and clinical characteristics of participants of lung cancer SNP-array GWAS**

**Table S26 Demographic and clinical characteristics of participants of ovarian cancer SNP-array GWAS**
