# Supplementary figures and images for: Boosting the Power of Rare Variant Association Studies by Imputation Using Large-scale Sequencing Population
Source: Genomics Proteomics Bioinformatics. 2025 Sep 17;23(5):qzaf084. doi: 10.1093/gpbjnl/qzaf084 (PMC13005946; doi:10.1093/gpbjnl/qzaf084)

## Lung cancer

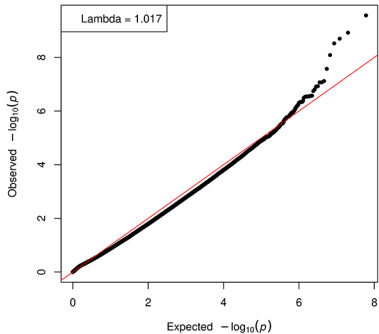

## Epithelial ovarian cancer

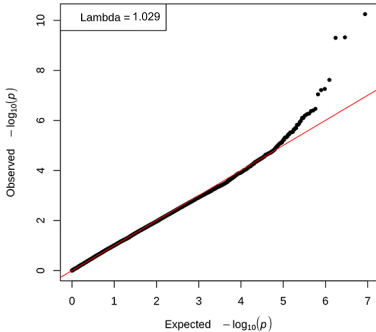

Supplement: qzaf084_Supplementary_Data [file qzaf084_supplementary_data.zip › Figure S10.pdf]

● TOPMed    ● HRC+UK10K

White

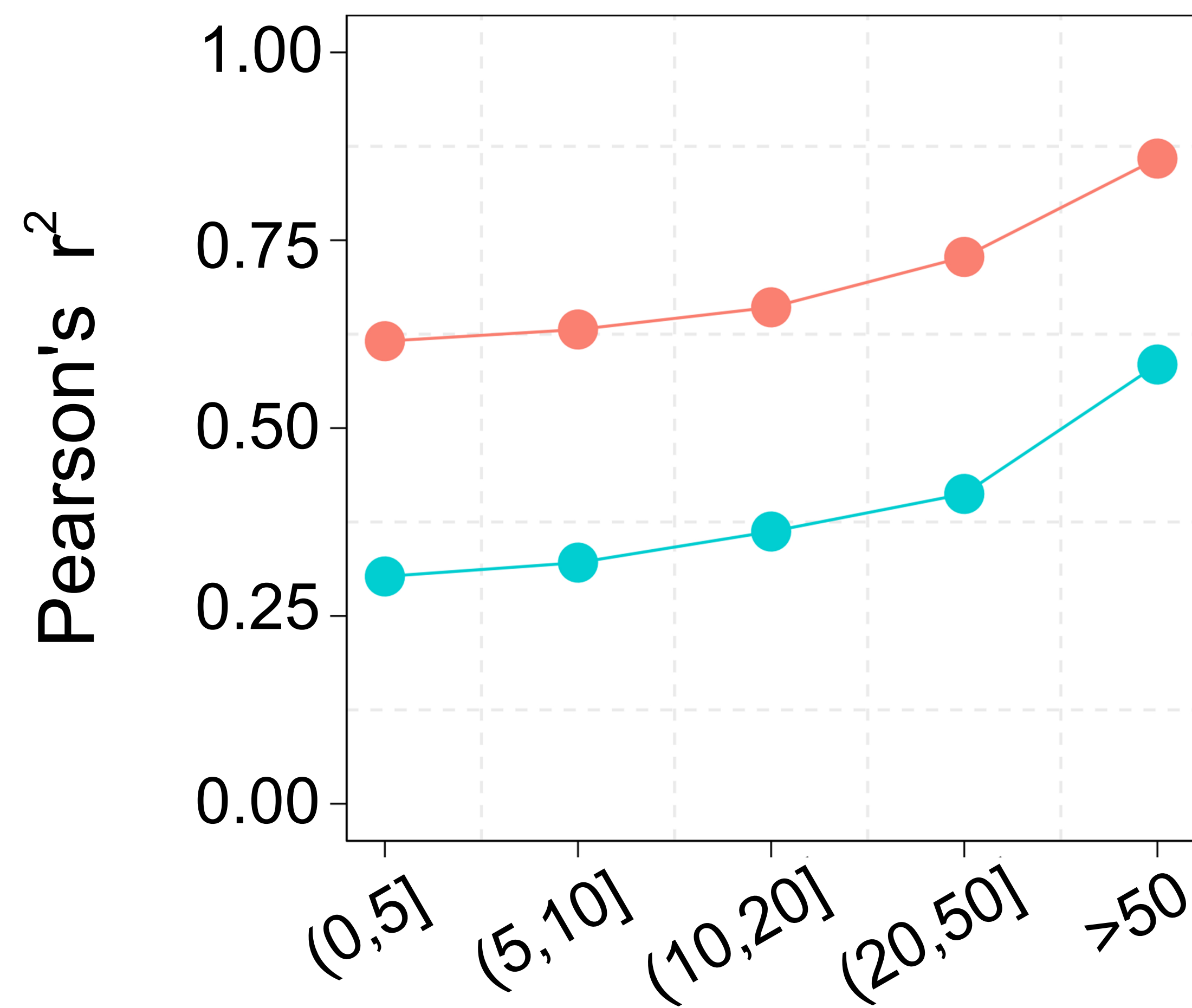

Asian

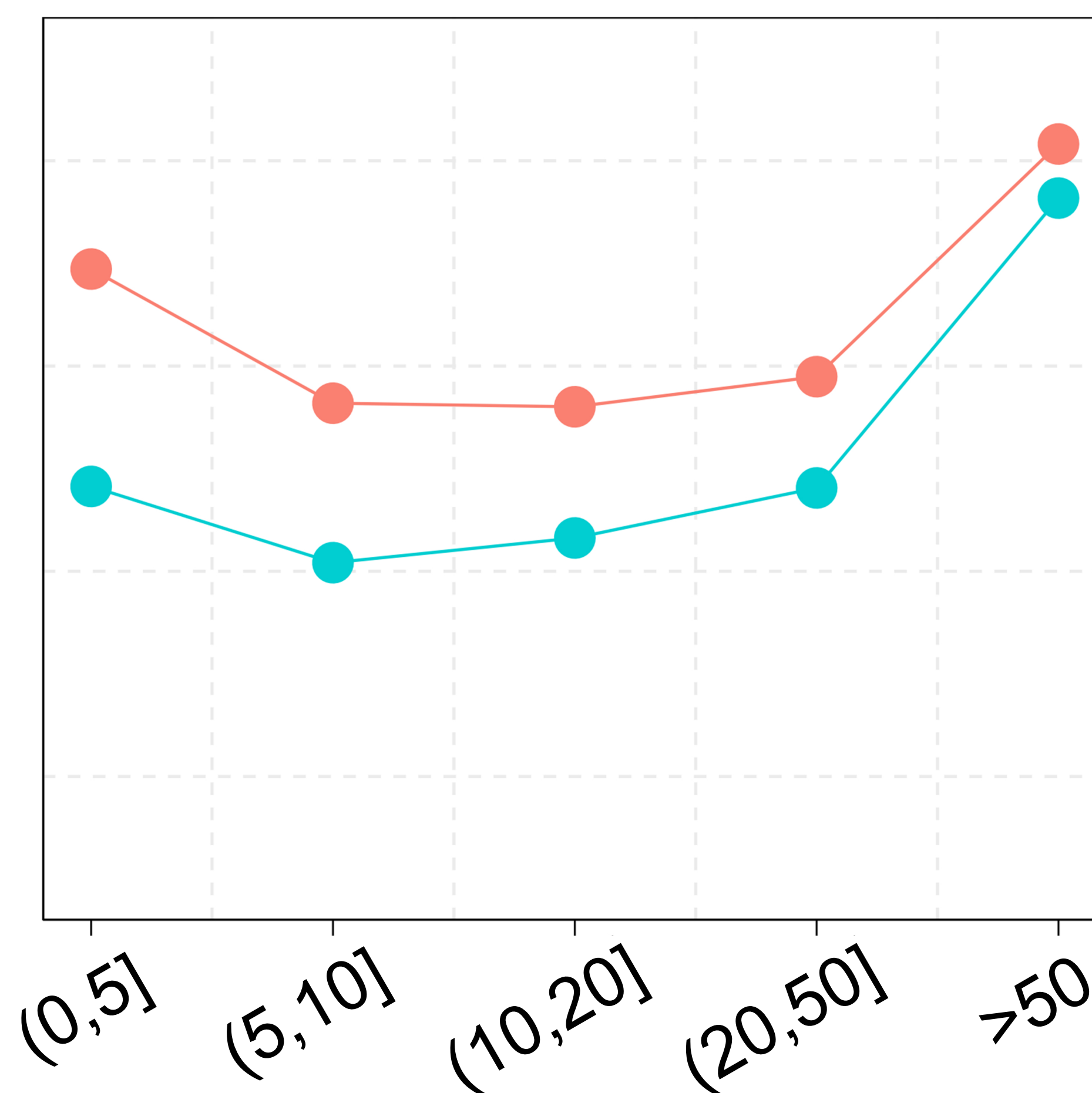

African

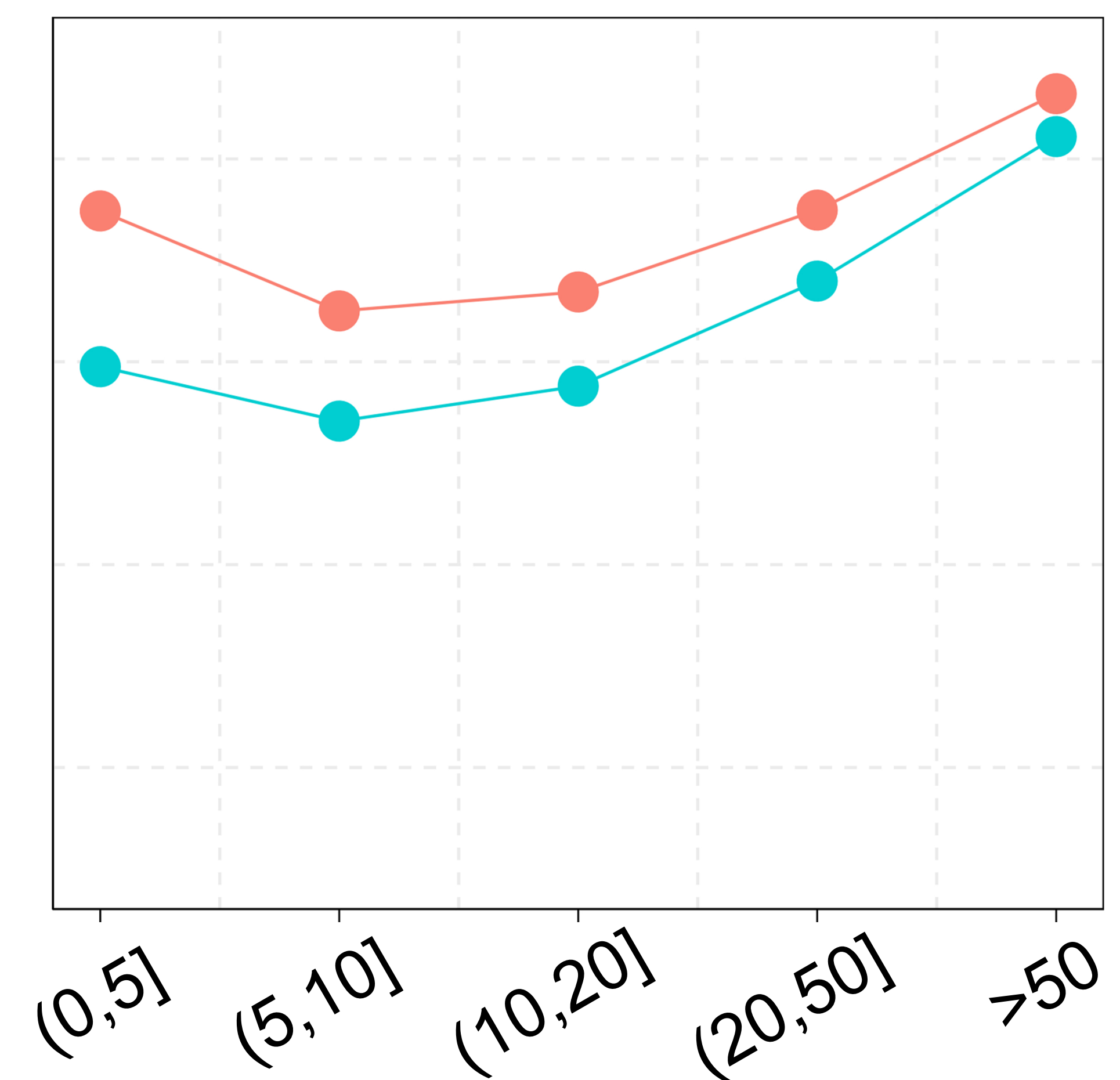

MAC intervals

Supplement: qzaf084_Supplementary_Data [file qzaf084_supplementary_data.zip › Figure S2.pdf]

## Single-variant tests

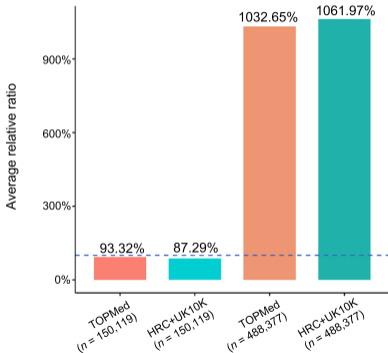

Supplement: qzaf084_Supplementary_Data [file qzaf084_supplementary_data.zip › Figure S3.pdf]

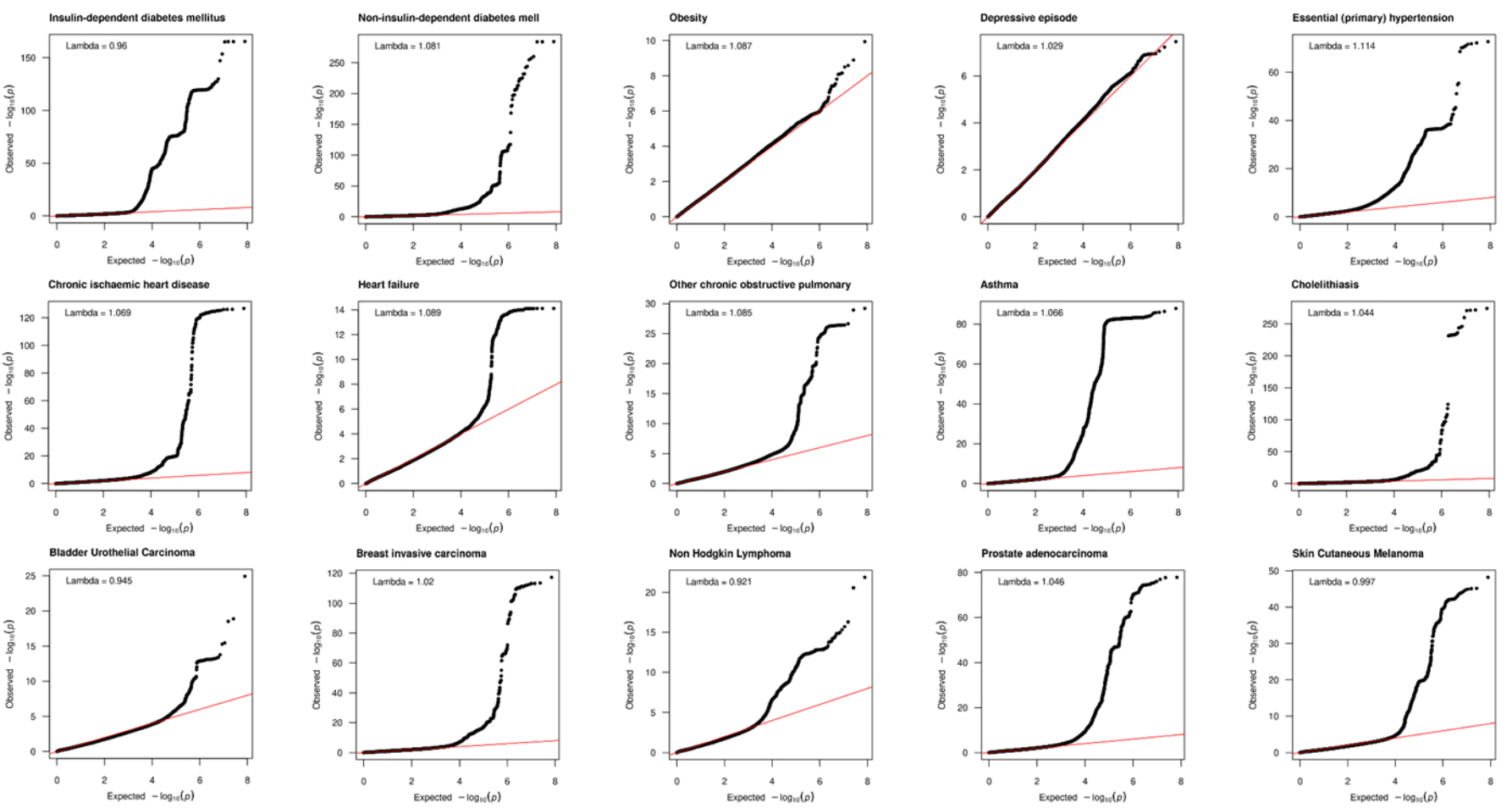

Supplement: qzaf084_Supplementary_Data [file qzaf084_supplementary_data.zip › Figure S4.pdf]

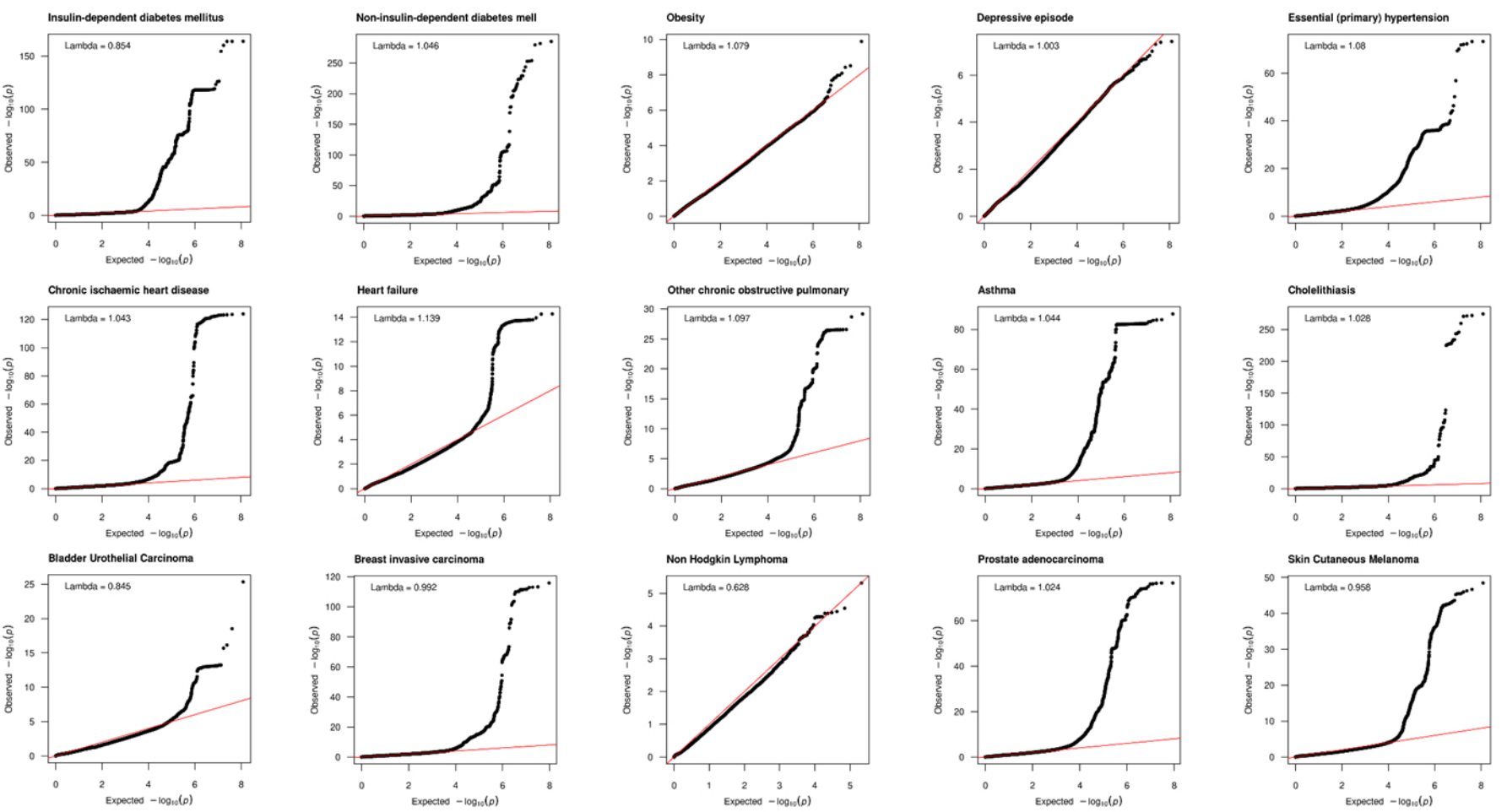

Supplement: qzaf084_Supplementary_Data [file qzaf084_supplementary_data.zip › Figure S6.pdf]

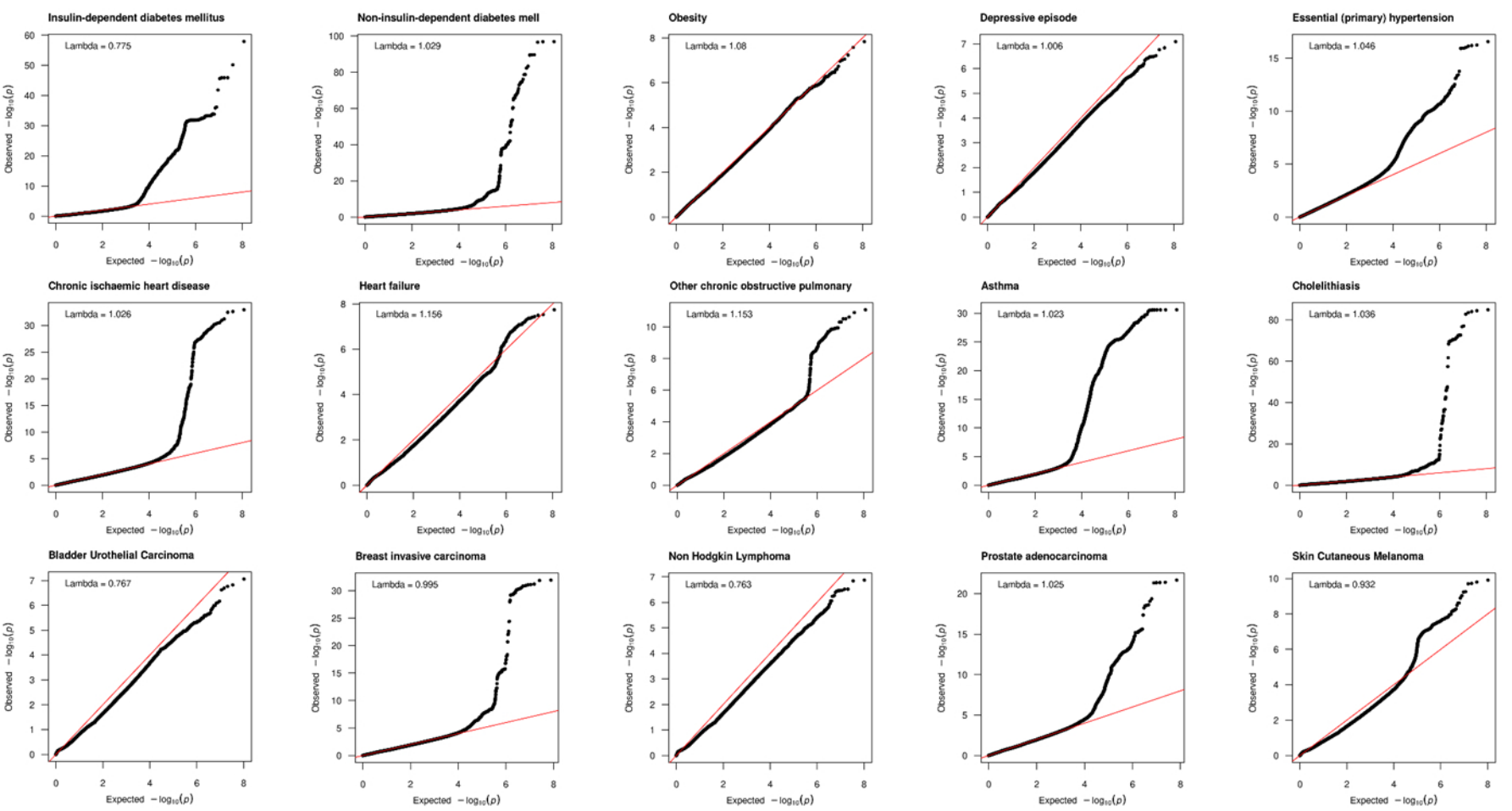

Supplement: qzaf084_Supplementary_Data [file qzaf084_supplementary_data.zip › Figure S8.pdf]

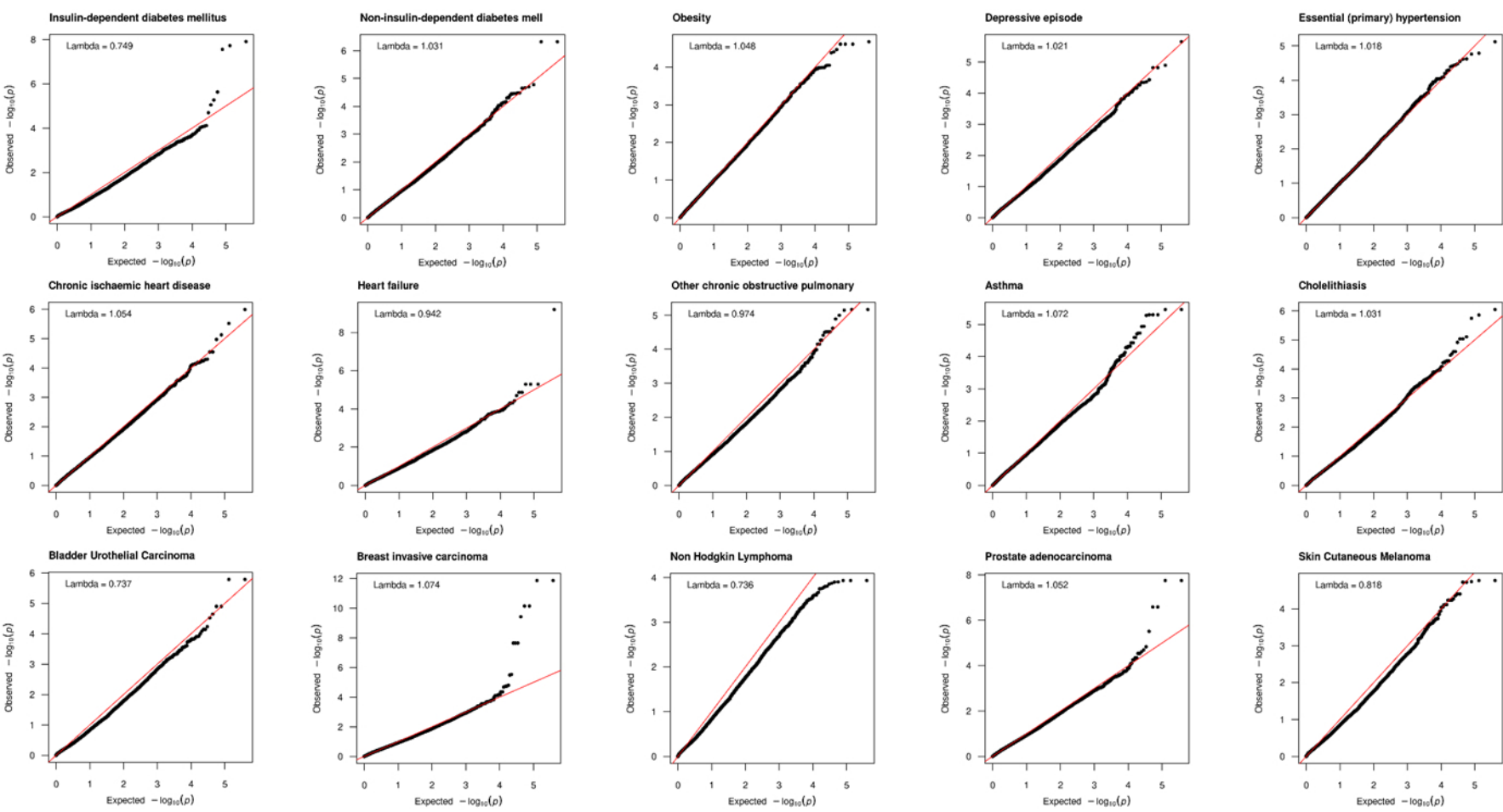

Supplement: qzaf084_Supplementary_Data [file qzaf084_supplementary_data.zip › Figure S9.pdf]
